# INSIG1 parallel substitution drives lipid/sterol metabolic plasticity mediating desert adaptation in ungulates

**DOI:** 10.1038/s42003-026-09523-z

**Published:** 2026-01-12

**Authors:** Xinmei Li, Ziyi He, Anguo Liu, Fanxin Meng, Xiao Zhang, Nana Li, Huan Liu, Yuyi Lu, Zhipei Wu, Huimei Fan, Xixi Yan, Nange Ma, Zhenyu Wei, Wei Wang, Xixi He, Kunyu Ma, Yu Jiang, Chao Tong, Bo Xia, Yu Wang

**Affiliations:** 1 Key Laboratory of Animal Genetics, Breeding and Reproduction of Shaanxi Province, College of Animal Science and Technology, Northwest A&F University, Yangling, Shaanxi China; 2 College of Animal Science and Technology, Northwest A&F University, Yangling, Shaanxi China; 3 School of Life Sciences, Arizona State University, Tempe, AZ USA; 4 Key Laboratory of Livestock Biology, Northwest A&F University, Yangling, Shaanxi China

**Keywords:** Molecular evolution, Conservation genomics

## Abstract

Desert ungulates, such as *Camelus bactrianus* and *Hippotraginae* antelopes, exhibit extraordinary adaptation to extreme environment. Deciphering these genetic adaptations is critical for understanding evolutionary resilience under climate change. Here, we generate a chromosome-level genome for domestic Bactrian camel and integrate comparative genomics analyses to uncover genomic adaptation in arid-desert ungulates. We find elevated molecular evolution rates with intensified positive selection among desert-adapted lineages. Convergent positively selected genes are mainly involved in energy metabolism, and ion transport and homeostasis. In addition, we identify further evidence reveals numerous parallel amino acid substitution genes associated with lipid/sterol metabolism, particularly cholesterol biosynthesis. Cross-species metabolomics reveal lower steroid-lipid levels in fasting camel serum, suggesting that genetic adaptation promotes metabolic trade-offs for desert survival. INSIG1 involved in cholesterol biosynthesis process emerge as a key candidate. Functional validation reveals that the INSIG1 mutation enhances lipid synthesis in energy-rich hepatocytes and promotes lipolysis during fasting in genome-edited male mice. Altogether, these findings highlight lipid/sterol plasticity as a cornerstone of desert adaptation, providing insights into breeding drought-resistant livestock and advancing therapeutic strategies for human metabolic disorders.

## Abstract (web summary)

A chromosome-level Bactrian camel genome and multi-omics comparisons reveal adaptive evolution and convergent selection in desert ungulates, highlighting lipid/sterol metabolism—especially INSIG1—as a key driver of metabolic flexibility for desert survival.

## Introduction

Deserts, covering approximately one-third of Earth’s terrestrial surface^1^, are among the most extreme habitats, characterized by limited precipitation, pole-end temperature, and food shortage^2^. These harsh conditions have driven the evolution of remarkable morphological and physiological adaptations in desert-dwelling species, making deserts natural laboratories for studying convergent evolution^3^. Amid the pressing threat of rapid global climate change, increasing attention has been given to research on adaptation to desert environments.

Previous studies have cataloged phenotypic adaptations in desert ungulates, including higher stable hematocrit^4–6^, specialized renal morphology^7–9^ and lipid storage and mobilization capacities^10^. Desert ungulates, such as Bactrian camels (*Camelus bactrianus*) and *Hippotraginae* antelopes (e.g., addax (*Addax nasomaculatus*), scimitar oryx (*Oryx dammah*), and gemsbok (*Oryx gazella*)) exemplify “endurers” capable of thriving in hyper-arid conditions^3^. These lineages diverged from their non-desert relatives (e.g., *Laminae* and *Reduncinae*) 15–16 million years ago, yet independently evolved convergent physiological traits, including adaptive heterothermy, enhanced water retention, and lipid-driven metabolic flexibility^11–14^. Similarly, desert-inhabiting species, such as fat-tailed sheep^15^, Merriam’s kangaroo rat (*Dipodomys merriami*)^16^, african fat-tailed gecko and fat-tailed gerbil^17^, exhibit analogous adaptations. Moreover, previous studies have suggested that the Arabian oryx utilizes water derived from fat metabolism, which may account for up to 24% of its total water requirement^3^. These traits provide a valuable framework for exploring how arid desert adaptations are shaped by evolutionary pressure. However, studies focusing on the genetic basis of traits that facilitate desert adaptation remain relatively scarce.

Genomic studies have revealed that the adaptation of ungulates and rodents to extreme desert environments involves positive selection genes related to energy homeostasis, salt metabolism, and maintenance of water balance^18–21^, including *AQP4*^22^ and *GPX3*^23^. However, most research has focused on single species or narrow traits, leaving the genetic basis of convergent arid-desert adaptations largely unexplored. Previous studies on convergent evolution have shed light on the genetic foundations of organisms inhabiting similar environments, such as mammalian subterranean^24^, aquatic adaptation^25^, and arctic adaptation^26^. Increased availability high-quality genome data of ungulates^11^, such as addax^12^, scimitar oryx^27^, and gemsbok^28^, provides resources for investigating the genetic underpinnings of remarkable adaptations to arid-desert environments.

Here, we generated de novo assembled chromosome-level genome for the domestic Bactrian camel using PacBio HiFi and Hi-C sequencing. By using publicly available genomic dataset, we analyzed 22 ungulate genomes to detect convergent evolutionary signatures in desert-adapted of *Camelus* and *Hippotraginae* lineages. By integrating genome-wide scan of positively selected genes (CPSG), identification of parallel amino acid substitution (PAAS), and cross-species metabolomics, we searched for genomic signatures of convergence underlying arid-desert adaptation. Specifically, ungulates inhabiting desert environments exhibit parallel substitutions at key regulatory sites of lipid and cholesterol metabolism, suggesting enhanced lipid/sterol metabolic plasticity that may facilitate survival under extreme conditions. To test this hypothesis, we performed functional validation in genome-edited mice and complementary in vitro assays, which provided strong support for the proposed adaptive mechanism. Collectively, these findings provide insights into breeding stress-resilient livestock and understanding human metabolic disorders.

## Result

### A new domestic Bactrian camel reference genome assembly and annotation

**Fig. 1:**
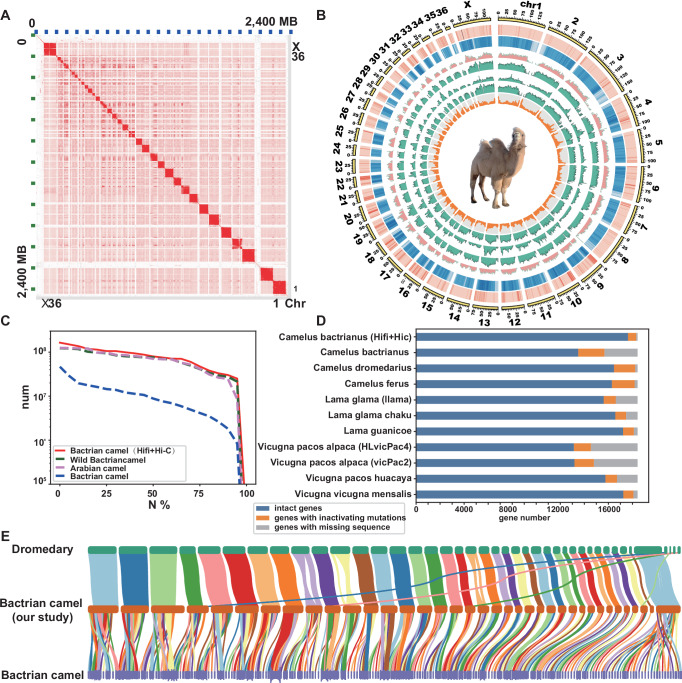
Chromosome-level genome assembly of domestic Bactrian camel. **A** Hi-C heatmaps among all chromosomes of the domestic Bactrian camel generated by Juicebox Assembly Tool^91^. **B** Chromosome architecture of the domestic Bactrian camel genome, using 1,000,000 bp windows. From the outer to inner rings: (1) chromosomes; (2) chromosomes length (Mb); (3) the number of genes; (4) the number of repeats; (5) the number of satellites; (6) the number of long interspersed nuclear elements; (7) the number of short interspersed nuclear elements; (8) the number of the long terminal repeats; (9) density of GC content. The domestic Bactrian camel illustration in this figure was entirely created by the authors using original artwork, with no third-party elements. **C** Comparison of camel genome between de novo assembly and reference in NCBI. Y axis shows scaffold sizes for which x percent of the assembly consisted of scaffolds of at least that size. **D** State of 18,430 ancestral placental mammal genes in Camelidae. **E** Syntenic relationships between de novo assembly in our study and published camel genomes.

To enhance the power to detect convergent genomic signatures, we firstly assembled a chromosome-level haplotype-resolved genome of the domestic Bactrian camel through a combination of PacBio circular consensus (HiFi) sequencing and high-throughput chromosome conformation capture (Hi-C) sequencing. All 36 autosome pairs and X chromosome are represented by chromosome-level scaffolds (Fig. 1A). Our assembly spanned 2.4 Gb consisting of 715 scaffolds with the contig N50 length of 58.8 Mb and scaffold N50 length of 79.9 Mb, which is better than existing camels’ assemblies (Fig. 1C, Table 1, Supplementary Data 1). The completeness of the new genome assembly that was reflected by the BUSCO value of 96.1%. The GC content was ~41.9%, and the repeats occupied 31.09% of the genome (Fig. 1B). There were more telomeric motifs and centromeric tandem repetitive sequences assembled compared with published reference camel genomes (Supplementary Fig. 1).

**Table. 1:** Summary statistics for the domestic Bactrian camel assembly

Assembly	De novo	Bactrian camel	Wild bactrian camel	Dromedary
Genome size	2.4 GB	2 Gb	2.1 Gb	2.2 Gb
Number of scaffolds	157	35454	2043	21070
N50 Scaffold length	79.9 Mb	8.8 Mb	70.3 Mb	70.3 Mb
Number of contigs	283	67434	4388	53085
N50 Contig length	58.8 Mb	139.1 kb	5.4 Mb	0.24 Mb
GC percent	41.9%	41%	41.48%	41.5%
Assembly	Chr	Scaffold	Chr	Chr

Subsequently, we employed de novo, transcriptome, and homology-based gene prediction methods to annotate the genomes. In total, 28,877 genes were annotated in the improved genome (Fig. 1B). To further compare assembly quality, we employed TOGA (Tool to Infer Orthologs from Genome Alignments), utilizing 18,430 ancestral placental mammal genes, to determine the highest number of intact genes in de novo genome among other genomes in *Camelidae* lineages (Fig. 1D; Supplementary Data 2). Our de novo genome assembly showed a highly conserved chromosomal synteny to dromedary and wild Bactrian camel, with chromosomes showing a one-to-one homologous relationship (Fig. 1E). Overall, we assembled a high-quality chromosome-level Bactrian camel genome, providing a robust foundation for advancing our understanding of arid-desert adaptation in ungulates.

### Convergent gene-wide patterns of molecular evolution in arid-desert adapted ungulates

**Fig. 2:**
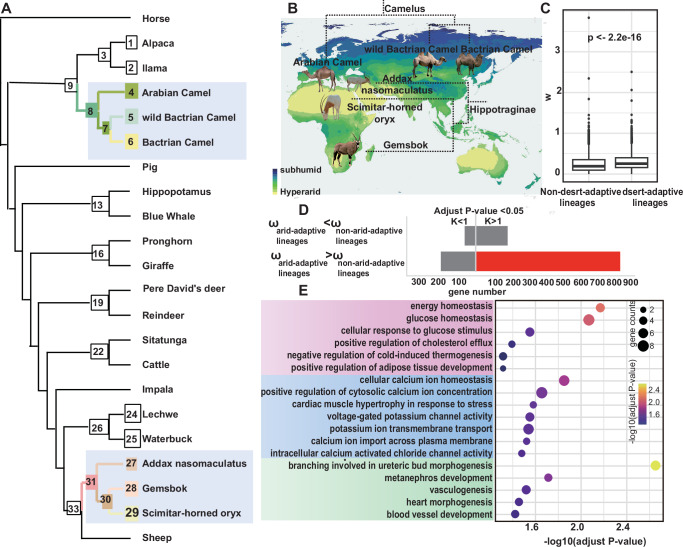
Identification of convergent positive selection genes and pathways. **A** Phylogenetic trees of the 22 species, shaded sections highlight extreme arid-desert-adapted *Camelus* and *Hippotraginae* lineages. Different numbers represent different inner and terminal nodes. **B** Geographical distribution of *Camelus*, the Bactrian camel (*C. bactrianus*) and Arabian camel (*C. dromedarius*), and the Hippotraginae species addax (*Addax nasomaculatus*), scimitar oryx (*Oryx dammah*), and gemsbok (*O. gazella*) in arid and semi-arid regions in the world. Different colors on the map represent different levels of drought. This map is adapted from the Global Aridity Index and Potential Evapotranspiration (Global-AI_PET) Database v3^128^, available under the CC BY-NC 4.0 license (https://creativecommons.org/licenses/by/4.0/). Photos of different species were adapted from Pexels (pexels.com) and are used under the terms of the Pexels License. *The dataset was reclassified based on Aridity Index (AI) thresholds to delineate arid and semi-arid zones*. **C** The boxplot shows the rate of gene-wise molecular evolution (ω, *dN/dS*) in arid-desert adapted lineages compared to non-arid-desert adapted branches. **D** The molecular evolutionary patterns of homologous genes in arid-desert adapted lineages. The foreground representing arid-adaptive lineages contrasts sharply with the background of non-arid-adaptive taxa. **E** Functional GO terms annotated by candidates for convergent positive selection genes.

To unravel the genetic basis of convergent desert adaptation, we analyzed 22 ungulate genomes, including *Camelus* and *Hippotraginae* lineages (Supplementary Data 3). A robust phylogeny was reconstructed (100% bootstrap support; Fig. 2A, B), confirming their divergence ~66 million years ago (Supplementary Fig. 2) and subsequent independent adaptation to arid environments (Fig. 2B). We obtained 12,489 high-confidence orthologous protein-coding genes by multiple genome synteny alignments, providing a foundation for detecting molecular convergence.

We initially conducted examination of selective pressures and direction to test the signatures of molecular evolution in two arid-desert adapted lineages. Arid-desert lineages (which defined as foreground) experienced higher gene-wide molecular evolution rates (*dN/dS*) compared to non-arid-desert lineages (which defined as backgrounds) (*p*-value < 2.2e−16, Wilcoxon rank-sum test, Fig. 2C), and coupled with a higher proportion of genes under intensification of positive selection (*p*-value = 0.009, Chisq test, Fig. 2D).

To further interrogated evolutionary pressures of orthologous genes across the *Camelus* and *Hippotraginae* lineages, HyPhy BUSTED-PH^29,30^ model was adopted to search for evidence of diversifying positive selection. We identified 538 convergent positive selection genes (CPSGs) after correcting for multiple testing (adjusted *p*-value (background) >0.05, adjusted *p*-value (foreground) <0.05, adjusted *p*-value (difference between background and foreground) <0.05) (Supplementary Data 4), which enriched pathways fall into three main functional categories: energy metabolism (e.g. positive regulation of adipose tissue development, glucose homeostasis, energy homeostasis, positive regulation of cholesterol efflux), ion transport and homeostasis (e.g. cellular calcium ion homeostasis, potassium ion transmembrane transport), development (e.g. metanephros development, vasculogenesis, heart morphogenesis) (Fig. 2E, Supplementary Data 5). Functional enrichments were largely consistent with those identified in previous studies, underscoring the pivotal role of certain biological strategies in shaping the adaptations to extremely harsh desert environment^3,31^. We obtained novel CPSGs like *GPLD1* and *ATP2B4*, suggesting adaptive shifts in energy utilization^32^ and blood pressure regulation^33^. Several genes such as *SLC24A3*, *LAMA5*, and *CRLS1* demonstrate not only convergent positive selection in desert-dwelling ungulates but also evidence of selection pressure in more distantly related lineages, specifically observed in desert foxes^31^. Altogether, the adaptive convergent genes and pathways offer significant insights into the complex processes underpinning adaptation to desert ecosystems, highlighting the intricate interplay between genetic evolution and environmental challenge.

### Convergent lipid/sterol metabolism adaptive evolution in arid-desert ungulates

**Fig. 3:**
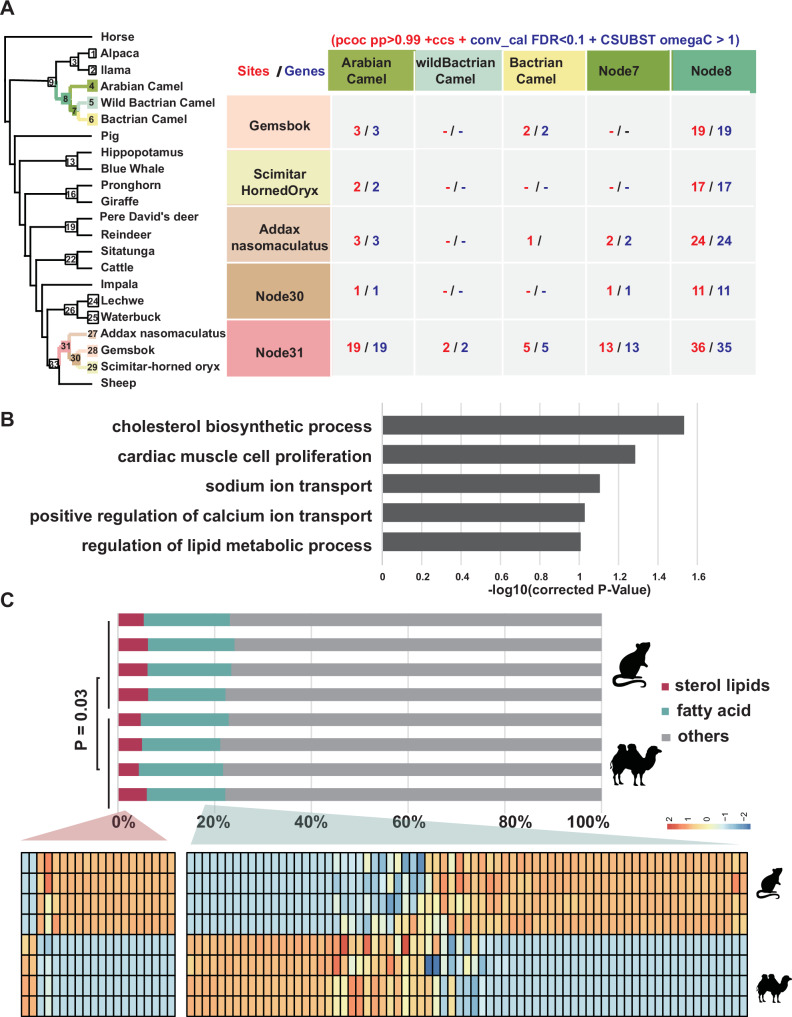
Cross-species comparisons reveal convergent desert-adaptive evolution of lipid/sterol metabolism. **A** The number of conservative parallel amino acid substitutions sites (red) and genes (blue) detected in 25 pairwise comparisons among *Camelus* and Hippotraginae lineages. **B** Functional GO terms enriched by parallel amino acid substitution genes. **C** Bar chart showing the percentage of super-class for identified metabolites in fatty acids, sterol lipids, and other metabolites of fasting camel and fasting mouse. Heatmap further showing the differences in identified fatty acid and sterol lipid metabolites super-class between camel and mouse. Red: sterol lipids; Green: fatty acid. *n* = 4 samples for male mice and camel. Animal silhouettes in (**C**) were obtained from PhyloPic (http://phylopic.org), which provides free silhouette images of organisms. The silhouettes used here are available for reuse under the Creative Commons Attribution 4.0 International License (CC BY 4.0; https://creativecommons.org/licenses/by/4.0/).

To robustly identify convergent evolutionary signatures, we performed pairwise convergence comparisons between each terminal branches and inner nodes across *Camelus* and *Hippotraginae* lineages (Supplementary Fig. 3), as adaptive variations can emerge at any evolutionary node or species in response to arid conditions. We then employed multifaceted approach to obtain candidates. Parallel amino acid substitution (PAAS) sites (we collectively refer to “parallel” and “convergent” substitution as PAAS) were primarily detected using PCOC^34^ (posterior probability [PP] >0.99), considering shifts substitution according the amino acid preference profile. To minimize false positives, we applied the extended Convergence at Conservation Sites (CCS) method^35^, filtering PAAS to retain those at moderately conserved positions. This stringent approach identified 427 candidate genes with 457 high-confidence PAAS sites. To assess background convergence noise, we performed seven control scenarios by swapping outgroups with target lineages, and yielded 568 genes with 635 substitutions (Supplementary Fig. 3, Supplementary Data 6). Similar to the trend of echolocation in cetaceans and bats^36–38^ and transitions to Viviparity in Cyprinodontiformes^39^, there was no excessive genome-wide convergence among arid-desert-adapted lineages compared to their outgroups, implying arid adaptation primarily targets specific loci. Subsequently, we further perform analysis to screen candidates with significant convergent signal at the gene level. By comparing observed substitution frequencies to neutral expectations in branch analysis, we confirmed 171 candidate genes exhibit statistical significance in arid-adaptation lineages relative to empirical controls after multiple correction analyses (Conv_cal method, Poisson test, Benjamini–Hochberg, FDR < 0.1)^38^ (Supplementary Fig. 3, Supplementary Data 6). To further detect the proteins evolved in similar directions among convergent lineages, we applied the CSUBST^40^ to calculate ωC (ωC = d*NC*/d*SC*) by distinguishing the effects of natural selection from genetic drift and phylogenetic errors. This method compares observed-to-expected ratios of nonsynonymous (d*NC*) and synonymous (d*SC*) substitutions across various phylogenetic branches. We revealed 157 candidates with ωC > 1 in arid-adapted lineages (Fig. 3A, Supplementary Fig. 3, 4, Supplementary Data 6), exhibiting significantly higher convergent evolutionary rates (ωC) than controls (Wilcoxon Rank Sum Test, *p*-value = 2.91e−16).

Despite strong signals of convergence, only ~7% of PAAS genes showed evidence of positive selection, likely due to pleiotropic constraints or transient or episodic selection regimes^41^. Examples include *PLEKHA7* act on salt-sensitive hypertension^42^, *LAMA5* associated with kidney disease^43^, *BUD13*^44^ and *ECI2*^45^ affect lipid/sterol metabolism process, and et al. To resolve this paradox, we employed the Mixed Effects Model of Evolution (MEME)^41^, which revealed that nearly all PAAS sites (93%) were under episodic positive selection (β + >α, *p* < 0.05; Supplementary Data 7). These candidates highlight loci under strong selection in arid environments, independent of phylogenetic or stochastic effects.

Functional analysis of PAAS genes revealed significant enrichment in cholesterol biosynthesis (Benjamini–Hochberg; adjust *p*-value = 0.0293) (Fig. 3B, Supplementary Data 8), a signal absent in control analyses (Supplementary Data 9). To validate these genomic findings, serum metabolomic profiling were conducted on fasting camels and mice, revealing a statistically significant elevation in the proportion of fatty acid-related metabolites, alongside a reduced proportion of steroid lipids in camel serum compared to mice (Fig. 3C, Supplementary Data 10, 11). The results are consist with previous studies that dromedaries have lower plasma cholesterol concentration and higher hepatic cholesterol than sheep and cattle^46,47^. Collectively, these results underscore lipid/sterol metabolic rewiring as a key adaptive strategy in desert ungulates, balancing energy storage during scarcity and rapid mobilization under fasting conditions, as a critical advantage for survival in extreme aridity.

### The parallel amino acid substitution in INSIG1 impact the lipids/sterols metabolism demonstrated by cell experiments

**Fig. 4:**
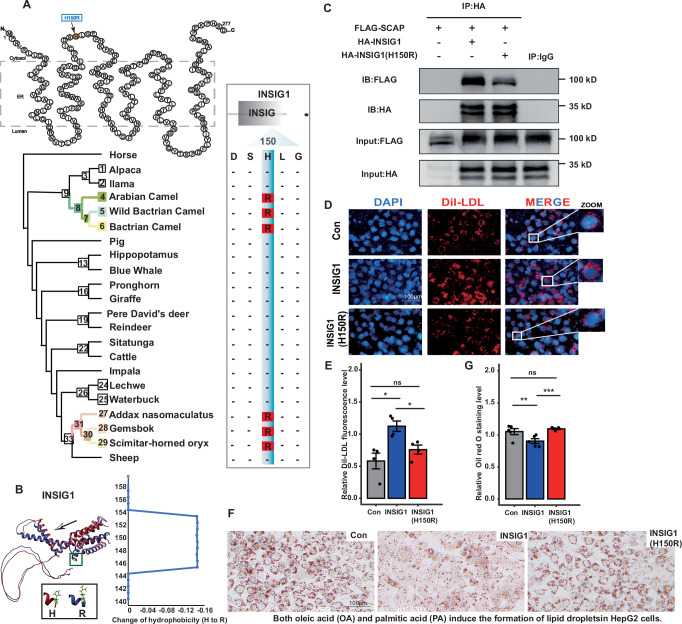
Adaptive convergent mutation in INSIG1 promote lipid and cholesterol accumulation. **A** Parallel substitutions at conserved sites of INSIG1 protein in *Camelus* and Hippotraginae lineages. **B** Mutation site in the 3D protein structures of INSIG1 was indicated in green. The red chains represent the normal protein structure, and the blue chains represent protein structure after parallel mutations. The arrows indicate the locations where structural changes occurred before and after the mutation. The hydrophobicity prediction of INSIG1. Amino acid substitution of H150R in INSIG1 reduces the amino acids hydrophobicity from 146 to 154. **C** Wild-type or mutant INSIG1 Co-IPed with SCAP were detected by western blotting. **D** Representative immunofluorescence images of Dil-LDL uptake in HepG2 cells from three independent experiments. Confocal microscopic images represent the fluorescence intensity of Dil-LDL (red) and DAPI (blue). Scale bars, 100 μm. **E** Relative DiI-LDL fluorescence levels were quantified in HepG2 cells transfected with empty vector (Con), INSIG1, or INSIG1(H150R). INSIG1 overexpression enhanced LDL uptake, whereas the H150R mutation significantly reduced LDL uptake compared with wild-type INSIG1. **F** OA/PA treated HepG2 cells were stained with ORO and lipid accumulation was visualized under a microscope at 100× magnification. Scale bars, 100 μm. **G** Relative ORO staining levels in the same groups showed increased neutral-lipid accumulation in cells expressing INSIG1(H150R) compared to wild-type INSIG1. Data are presented as mean ± SEM from at least three independent experiments All experiments were repeated at least three times, and representative data are presented. Values and error bars represented means and ±SEM from multiple independent biological replicates (DiI-LDL (*n* = 4 biologically independent experiments); ORO (*n* = 5 biologically independent experiments)). Asterisks indicate significant differences. *P ≤ 0.05, **P ≤ 0.01, ***P ≤ 0.001, NS: not significant by one-way ANOVA with Tukey’s post-hoc test.

Among candidates in cholesterol biosynthetic process, INSIG1 and NPC1L1 pronounced the strongest convergent signals, with PAAS at highly conserved sites (site-wise posterior probability of convergence >0.95; Supplementary Fig. 5). *INSIG1* regulates lipid and sterol synthesis via negative feedback, while *NPC1L1* mediates cholesterol transport^48,49^. PAAS in INSIG1 and NPC1L1 altered the 3D-structure and hydrophobicity (Fig. 4A, B, Supplementary Fig. 6). In addition, *INSIG1* and *NPC1L1* had lower expression levels in camel liver tissue using available data comparing to cattle, pig and human (Supplementary Fig. 7, Supplementary Data 12). Hence, the parallel substitutions caused the influence on characteristics of proteins, suggesting their involvement in adaptation to severe drought.

Previous research has demonstrated that a single amino acid mutation, D205A, plays a critical role in the function of INSIG1 proteins in regulating cholesterol homeostasis^50^. Our study found INSIG1 (H150R) substitution under positive selection, located within its functional domain, influenced the stability of the proteins (∆∆G = −0.425) and disrupted binding pockets (Supplementary Fig. 8). INSIG1 can trigger to bind the SREBP cleavage–activating protein (SCAP) to reduce synthesis of cholesterol, fatty acids^51^. To assess the functional effect of mutations on the binding efficiency of INSIG1 to SCAP, we co-transfected with Flag-SCAP in Human liver cancer cell line (HepG2) cells. The results of CO-IP revealed that mutant INSIG1(H150R) blocked binding of SCAP (Fig. 4C, Supplementary Fig. 9, Supplementary Data 13). To directly assess the impact of mutation on lipid/cholesterol synthesis, we constructed lipid accumulation model using HepG2 cells (hepatoblastoma cell line) treated with the free fatty acid palmitic acid/oleic acid in vitro. Oil Red O (ORO) results showed that the INSIG1(H150R) mutation significantly promoted lipid accumulation in hepatocytes compared with wild-type INSIG1 (Fig. 4F, G; Supplementary Data 14). We then constructed wild-type and mutant INSIG1 plasmids, transfected HA-INSIG1 and HA-INSIG1(H150R) alone, and found that the mutation resulted in lower LDL uptake levels compared with the wild-type in HepG2 cells (Fig. 4D, E; Supplementary Data 15). By decoupling lipids/sterols homeostasis from feedback inhibition, desert ungulates may optimize energy reserves-a vital strategy for surviving unpredictable food scarcity in arid ecosystems.

### The *Insig1*^*H132R/H132R*^ mice verified mutation promoted lipid/sterol synthesis and mobilization

**Fig. 5:**
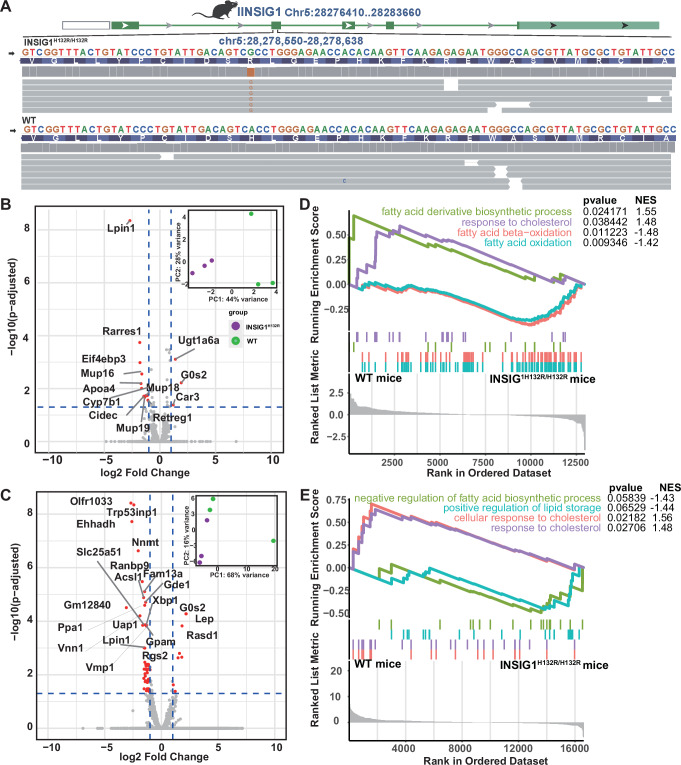
Functional experiments verified INSIG1 mutation that contribute to lipid/sterol synthesis and mobilization. **A** *Insig1*^*H132R/H132R*^ gene-edited mice strategies. Human H150R and mouse H132R are orthologous at the same conserved INSIG1/Insig1 site; numbering is species-specific. Animal silhouettes obtained from PhyloPic (http://phylopic.org), which provides free silhouette images of organisms. The silhouettes used here are available for reuse under the Creative Commons Attribution 4.0 International License (CC BY 4.0; https://creativecommons.org/licenses/by/4.0/). Differentially expressed genes in liver (**B**) and visceral fat (**C**) of homozygous and wild-type mice. PCA of the liver and visceral fat transcriptomic datasets between mutant and wild-type mice. *n* = 3 samples of homozygous and wild-type male mice. GSEA analysis showed enrichment of DEGs in liver (**D**) and visceral fat (**E**) between wild-type mice and *Insig1*^*H132R/H132R*^ mice. NES, normalized enrichment score.

To better understand the role of the INSIG1 mutation in vivo, we generated a mice model through point mutation employing CRISPR-Cas9 gene editing. The gene-edited mice were sequenced and confirmed no evidence for off-target effects (Fig. 5A). There was no statistically significant difference between cholesterol and triglyceride concentrations in serum and liver of mutant and wild-type mice after fasting for 14 h in our study (Supplementary Fig. 10A, Supplementary Data 16). Consistent with the Insig1^−/−^ and Insig2^−/−^ mouse phenotype, the levels of plasma cholesterol and free fatty acids showed no change^52^. We further performed the liver and visceral fat transcriptome sequencing of mutant and wild-type fasting male mice. Sterol regulatory element-binding proteins (SREBPs) as master regulators of cholesterol synthesis, uptake, and lipid metabolism, exhibiting marked downstream effects in the edited mice^53–55^: LDLR, SCD1, and PCSK9 were reduced in liver; ACACB and ACLY were reduced in visceral adipose; and LPIN1 increased in both tissues (Supplementary Fig. 11). The result of differentially expressed genes (DEGs) were significantly enriched pathways related to cholesterol, lipid and glucose metabolism process (Supplementary Fig. 12A, B). Noticeably, the mutant mice markedly enhanced the expression of genes responsible for lipid/sterol degradation, such as *LPIN1*^51,56^*, APOA4*^57,58^, *PDK4*^59^ and et al., as well as CPSG like *ADCY5*^60^ and *CRLS1*^61–63^, while genes as *GOS2*^64,65^ and *PNPLA3*^66^ related to lipid/sterol synthesis were significantly down-regulated (Fig. 5B, C, Supplementary Data 17). We further validated differential expression of PLIN1 and G0S2 between wild-type and mutant mice by RT-qPCR, and the results were concordant with the transcriptomic data (Supplementary Fig. 13, Supplementary Data 18, 19). Consistently, comparing to wild-type mice, the result of gene set enrichment analysis (GSEA)^67^ of liver and visceral fat revealed significantly upregulated enrichment in “fatty acid oxidation”, “negative regulation of fatty acid biosynthetic process” in the *Insig1*^*H132R/H132R*^ mice, while downregulated “response to cholesterol” and “fatty acid derivative biosynthetic process” (Fig. 5D, E). Given previous studies and our own evidences both point out the effect of *INSIG1* on metabolism process, we further investigated the downstream influence on the liver metabolite using liquid chromatography–mass spectrometry (LC-MS). We found differential metabolites between *Insig1*^*H132R/H132R*^ and wild-type mice associated with lipid/sterol metabolism, including Cholesterol sulfate, 15-KETE, Aminoadipic acid and et al. (Supplementary Fig. 10B, Supplementary Data 20). These results align with transcriptional profiles, suggesting INSIG1 H150R mutation accelerates lipid mobilization during energy scarcity as a key survival strategy for desert ungulates.

## Discussion

Comparative genomic analyses focusing on convergent evolution have emerged as a powerful approach for elucidating the genetic underpinnings of species-specific traits and adaptations to environmental challenges^68–70^. In this study, we de novo assembled a chromosome-level, haplotype-resolved Bactrian camel genome and conducted an analysis of convergent evolution between the *Camelus* and *Hippotraginae*, the lineages of ungulates adapted to arid desert environments. Comparative genomics, along with cross-species metabolomic and comparative transcriptomic analyses, confirmed that lipid/sterol metabolism is crucial for arid-desert adaptation. Additionally, experiments and gene-editing of *Insig1*^*H132R/H132R*^ mice demonstrated that a single amino acid substitution site putatively underlies the enhanced synthesis and mobilization of lipids/sterols in vivo.

Convergent evolutionary events can be influenced by various biological factors like incomplete lineage sorting (ILS) and introgression^71^, background noise resulting from theoretical models^72^ and random convergence^36^ can all compromise the fidelity of convergent signatures. To enhance the robustness in pinpointing adaptively convergent candidates, we implemented a multi-layered analytical pipeline. Firstly, PCOC^34^ and CCS approaches^73^ were combined to identify high-confidence parallel amino acid substitutions (PAAS) sites. Seven non-arid ungulate lineages served as empirical controls to diminish background noise. Finally, gene-level significance was assessed via Conv_cal^38^ and CSUBST^40^, ensuring retained candidates reflect arid-specific adaptation rather than random drift. While no method can fully eliminate false positives, this integrative approach significantly enhances confidence in our findings.

Lipid metabolic plasticity is a recurring theme in species adapting to extreme environments. High-altitude yak^74^, arctic muskox and reindeer^26^, hibernating species like bears^75,76^ and cave-dwelling creatures like cavefish^77^, have developed significant lipid metabolic adaptability. Strong lipid storage and rapid mobilization capabilities are putatively conserved strategies to meet energy demands in extreme environments^78–80^. Similarly, desert animals face dual challenges of energy scarcity and water deprivation^15,31,81^. Our study revealed that cholesterol biosynthesis exhibits the strongest convergent signal (Supplementary Fig. 5), and highlighted two potential adapted convergent genes *INSIG1* and *NPC1L1*, essential for maintaining cholesterol balance^48,49,82^. For arid-desert adapted ungulates, boosting lipid metabolism is essential not only for fulfilling energy requirements but also for coping with extreme water scarcity. As the lipid oxidation yields more metabolic water than protein and carbohydrate^83^. And the cholesterol levels are crucial for maintaining internal water balance in camels under dehydration conditions^84^.

Furthermore, the convergent substitution of single amino acid in INSIG1 stood out driving lipid/sterol metabolism shift suggested by both in vitro and in vivo experiments. Notably, the mutation of INSIG1 has also been found in other species known for pronounced fat deposition capabilities, in which hibernating bear and polar bears exhibit elevated cholesterol levels (Supplementary Fig. 14)^85–87^. Our results revealed significant upregulation of *PDK4* in the visceral fat of *Insig1*^*H132R/H132R*^ mice, *PDK4* plays a key role in metabolic adaptability during hibernation by facilitating extensive lipid storage and utilization^59,80^. Our finding provided additional evidence of *INSIG1* as a promising therapeutic target for combating diseases related to lipid/sterol dysregulation. This observation is in concordance with previous studies illustrating that single amino acid variations can exert the profound biological impact. For instance, in cavefish, coding mutations in MC4R enhanced appetite and starvation resistance^78^, P211L mutation in INSRA increased weight and insulin resistance^88^. The Q247R mutation in RETSAT provided novel insights into the genetic mechanisms of hypoxia adaptation and offers new therapeutic targets for pulmonary arterial hypertension and right ventricular hypertrophy^89^. Therefore, we propose that PAAS sets may represent loci of significant biological relevance.

Additionally, we detected convergently positively selection genes related to lipid/steroid metabolism, such as *GPLD1* and *LANCL2*. *GPLD1* is significantly differentially expressed across species and possesses conserved parallel amino acid substitution sites (pp > 0.99), although it was filtered out in the gene-wise level pipeline (Supplementary Fig. 15). Collectively, our study furnishes additional evidence supporting the indispensable role of lipids/sterols in arid-desert adaptation.

Here, we employed comparative genomics approaches to further elucidate the potential molecular mechanisms underlying the adaptation to extreme desert environments. Future integration of techniques such as ChIP-seq and ATAC-seq with comparative genomics to identify regulatory variations and elements is essential for studying and understanding environmental adaptation. Despite these limitations, by integrating multi-omics approaches, including cross-species comparative genomics, transcriptomics, and metabolomics, our study elucidates the genetic underpinnings of ungulates’ adaptation to arid-desert environments, with a particular focus on the pivotal role in lipid/sterol metabolism. Importantly, we combined multi-omics approaches with cellular assays and gene-edited mouse models, which allowed us to directly validate the functional consequences of key mutations on lipid/sterol metabolism. The in vitro and in vivo experiments provide mechanistic evidence linking specific variants to altered INSIG1-SCAP interactions and downstream metabolic regulation. Moreover, these findings highlight potential gene-editing targets for breeding stress-resistant livestock, inform strategies for developing drought-resistant species under climate change, and deepen our understanding of human metabolic disorders, including obesity, diabetes, and non-alcoholic fatty liver disease (NAFLD).

## Method

### Samples collection

In this study, the tissue samples used for genomic sequencing were collected from a healthy female domestic Bactrian camel. A healthy adult female domestic Bactrian camel (Camelus bactrianus) was sourced from Inner Mongolia with veterinary certification of good health. The animal was not used in prior experimental procedures and was maintained under standard husbandry before tissue collection.

For terminal sampling, the camel was deeply anesthetized with an intravenous combination of 0.3 g xylazine and 0.9 g ketamine, followed by euthanasia via 59.6 g sodium pentobarbital administered intravenously. Death was confirmed by loss of corneal reflex and cardiac arrest before tissue sampling. Whole blood (~200 mL) was collected via jugular venipuncture into EDTA-coated tubes for genomic DNA extraction. After collection, the blood samples were immediately stored at −80 °C until further processing. Within ~10–15 min post-mortem, liver tissue (more than 500 mg) was dissected, rinsed briefly in ice-cold phosphate-buffered saline (PBS), snap-frozen in liquid nitrogen, and stored at −80 °C until Hi-C library construction. All samples were subsequently shipped on dry ice to Novogene (Beijing, China) for genomic DNA extraction, Hi-C library preparation and sequencing.

For the mouse tissue experiments, male wild-type and genome-edited mice aged 8–12 weeks were bred and housed under specific-pathogen-free conditions at the Animal Core Facility of Northwest A&F University (Shaanxi, China; certificate no. SCXK [SHAAN] 2017-003), with controlled temperature and humidity, a 12 h light/dark cycle, and free access to standard chow and water. For terminal sampling, mice were deeply anesthetized with isoflurane inhalation followed by euthanasia via cervical dislocation. Death was confirmed before dissection. Liver and subcutaneous epididymal white adipose tissue (eWAT) were rapidly collected within 5–10 min post-mortem, rinsed briefly in ice-cold PBS, blotted dry, snap-frozen in liquid nitrogen, and stored at −80 °C until further analyses.

For metabolomics analysis, additional blood samples were collected from adult male domestic Bactrian camels (*n* = 4) sourced from Inner Mongolia with veterinary certification of good health and adult male mice (*n* = 4) from the Animal Core Facility of Northwest A&F University (Shaanxi, China). All animals were fasted for 12 h prior to blood collection (food withdrawn, water available ad libitum). Camel blood was drawn by jugular venipuncture; mouse blood was collected via the retro-orbital plexus under light isoflurane anesthesia using sterile needles. Blood samples were allowed to clot at room temperature for 30 min and then centrifuged at 1500 × *g* for 10 min at 4 °C to obtain serum. The resulting serum was aliquoted and stored at −80 °C until metabolomic profiling.

We have complied with all relevant ethical regulations for animal use. All procedures were reviewed and approved by the Northwest A&F University Animal Care Committee (Approval No. DK2022049). We took all necessary precautions to ensure animal welfare during tissue collection, and all procedures adhered to ethical guidelines for animal care. To ensure compliance with experimental standards and animal welfare, all operations followed the guidelines of the Animal Ethics Committee, and all sampling processes were carried out under the supervision of trained personnel, ensuring both animal welfare and the reliability of experimental data.

### Genome sequencing

Genomic DNA (gDNA) extraction as well as CCS/PacBio long-read library preparation and sequencing, and Hi-C library preparation and sequencing were performed by Novogene (Beijing, China) according to the vendor’s standard operating procedures.

Genomic DNA (gDNA) was extracted from the blood for HiFi reads generation. Genome sequencing was performed using CCS (Circular Consensus Sequencing Mode) on the Pacbio Sequel II platform. Finally, 37.59 Gb, 38.86 Gb, and 40.32 Gb of CCS reads in 3 cells were yielded, respectively. In addition, liver tissue collected from the same camel was used for Hi-C library construction, and 206.173 G raw data were obtained using the Illumina sequence. To ensure the quality of information analysis, raw reads must be filtered by the following criterion: (1) Remove the reads pair with adapter; (2) Remove paired reads that contain more than 10% N content in a single-end read. (3) Remove paired reads that contain low-quality (Q ≤ 5) bases of more than 50% of the read length ratio. We ended up with 204.135 G of clean data, which were used for subsequent analysis.

### Genome assembly and annotation

We first extracted fasta from bam files by “samtools fasta”, and then provided merged fasta to HiFiasm^90^. We used *generate_site_positions.py* in Juicer^91^ to pre-calculate the position of the enzyme restriction sites with respect to the draft genome sequence (generate_site_positions.py MboI camel draft.fa). Identifying the precise location of each restriction site within the genome is crucial for accurately mapping the fragments back to the reference genome and ensuring that the observed interactions between genomic regions are based on accurate positional relationships. And a draft assembly was obtained by running Juicer with the raw fastq files from sequencing a Hi-C library (juicer.sh –g draft –s MboI –z draft.fa –y draft_MboI.txt –p assembly). Script run-asm-pipeline.sh in 3D de novo assembly (3D-DNA) pipeline combined with a review step in Juicebox Assembly Tools^91^ to yield highly accurate assemblies. The candidate 157 chromosome-level scaffoldings were manually reviewed using Juicebox assembly tools until the overall heatmap matched the characteristics of chromosome interaction. Finally, we got three preliminary assemblies including one monoploid assembly and two haploid assemblies, which spanned 2.4 Gb (Haploid), 2.2 Gb (Haploid-1), and 2.3 Gb (Haploid-2). The transposable elements were identified using RepeatMasker (v4.1.1)^92^ (RepeatMasker -pa 20 -engine ncbi -species Camelidae -xsmall -s -no_is -cutoff 255 -frag 20000 -dir. -gff de novo genome). As a complement, tandem repeats composed of a motif occurring twice or more were predicted using TRF v4.09^93^. To annotate telomeres, we searched for clusters of (AACCCT)n repeats throughout the genomes using the Telomere Identification toolkit (Tidk, v0.2.0) (https://github.com/tolkit/telomeric-identifier) and RepeatMasker. The completeness of the de novo genome was assessed by Benchmarking Universal Single-Copy Orthologs (BUSCO) with “mammalia_odb10” ^94^.

To perform gene annotation on the de novo genome, we integrated three complementary approaches: ab initio prediction, transcriptome-guided annotation, and homology-based inference. For ab initio prediction, AUGUSTUS (v3.4.0)^95^ was trained using an iterative optimization protocol, wherein initial gene models derived from the Bactrian camel reference annotation, wherein initial gene models derived from the Bactrian camel reference annotation^20^ were randomly partitioned into training (80%) and test (20%) sets, ensuring a minimum of 200 loci for parameter calibration. After five rounds of cross-validation to refine species-specific parameters (e.g., splice site motifs, codon usage), the optimized model was applied to annotate the full genome. We collected publicly available transcriptomic data from 38 samples of six adult Bactrian camel tissues (liver, lung, adipose, renal medulla, muscle, and pancreas) from NCBI for transcriptome annotation. The corresponding SRA accessions are SRP247453^96^, SRP014573, SRP122491, and SRP148535^97^ (Supplementary Data 21). The transcriptome data were first aligned to the genome via HISAT2 (v2.2.1), followed by genome-guided assembly with Trinity (v2.15.1)^98^ using parameters --genome_guided_bam all.sort.bam --max_memory 50G --genome_guided_max_intron 10000 --CPU 6. For homology-based annotation, protein sequences from three camelid species (*C. dromedarius*, *C. ferus*, *C. bactrianus*) were aligned to the genome using miniprot (v0.7) with thresholds of ≥70% sequence identity and coverage^98^. The three sets of gene models were integrated through EvidenceModeler (EVM v1.1.1)^99^, prioritizing transcriptome-derived evidence, followed by homology and ab initio predictions. Specifically, we assigned weights of 5, 3, and 2 to the TRANSCRIPT, PROTEIN, and ABINITIO_PREDICTION evidence, respectively, and then performed the integration using the following script: evidence_modeler.pl --genome genome.fa --weights weights.txt --gene_predictions denovo.gff --protein_alignments proteinprediction.gff --transcript_alignments transcripts.fasta.transdecoder.genome.gff3 evm.out. Finally, annotation completeness was validated using TOGA (v1.0)^100^ against 18,430 conserved mammalian orthologs, achieving >95% intact gene recovery, thereby ensuring high-confidence gene structures for downstream analyses.

### One-to-one orthologous genes and genome-scale phylogeny

Orthologous genes between 22 species (Supplementary Data 3) were identified by Multiple genome synteny alignments. Previous evaluations on simulated data have indicated that the differences are minimal in gene region while whole-genome alignments^101,102^. Over closer evolutionary distances, these alignment tools perform very well across all annotated regions.

The cattle (Bos taurus, UCD1.2) genome as reference, we utilized the LAST (version last1205) for pairwise genomic alignment which means the genome of each species is compared with the reference genome. In this stage, we first tried to find suitable score parameters for aligning the given sequences (lastdb -P0 -uMAM4 -R00; last-train -P0 --revsym --matsym --gapsym -E0.05 -C2 index_name species.fa > species.mat) and align similar sequences (lastal -m 100 -E 0.05 -C2 -P20 -p species.mat index_name species.mat ruminant.species.fa | last-split -m1 > result.maf), after that, multiple alignment files were gained using last-split and maf-swap toolkit. The following step is merging multiple alignment files (MAF) through Multiz (Version 11.2). maf_project was employed to extract MAF blocks that include a given reference sequence or species.

In order to obtain one-to-one orthologous genes, we converted the MAF files into a list format, where each line corresponds to a genomic position and the aligned sequences from each species at that position. Finally, we extracted the CDS regions and related positional information from the cattle’s GFF file. Based on the base correspondence obtained from the previous step, we extracted the gene sequence information for each species at these positions. If multiple transcripts for gene in the same species were recovered, we kept the longest. For each gene, we generated a file containing the FASTA sequences from all 22 species. we adopt in-house Perl scripts to gain 18,719 orthologous genes. After that, we used MACSE v2^103^ to further align and split each orthologous exon, taking into account the characteristics of protein coding, such as using explicit codon evolution models and considering insertions, deletions, and reading frames. Finally, totally 12489 orthologous protein-coding genes were retained for further analysis after removing the low mass alignment.

We also obtained Fourfold Degenerate Synonymous Site (4DTv) to construct a phylogenetic tree. The following step is that using Gblocks^104^, and trimal^105^ methods trim 4DTv to capture the conserved blocks/segments that may be more reliable regions from which to compare evolutionary rates. Finally, the phylogenetic tree was inferred by IQ-TREE^106^ (iqtree -s 4Dsite.fa-gb -nt 4 -bb 1000 -m TEST) which apply a fast and effective stochastic algorithm by maximum likelihood. Phylogenetic relationships were consistent across different approaches. To evaluate precisely the divergence times in ungulates, our whole genome alignments across 22 species and utilized the MCMCtree program in PAML (v4.9)^107^ (http://abacus.gene.ucl.ac.uk/software/paml.html) to infer the divergence times. Fossil dates were obtained from the Timetree website (http://www.timetree.org/) and previously published articles^11,108^.

### Molecular evolution analyses

We used coding sequences extracted from the whole-genome alignment to identify evidence for positive selection. Each coding sequence was then realigned using the “refineAlignment” in MACSE v2.06^103^, which generated both nucleotide and amino acid alignments for each coding region. We further adapted the “exportAlignment” to replace all frameshifts with gaps, convert stop codons at the end of sequences into gaps, and substitute all internal stop codons with “N”.

We defined the two arid-adaptive lineages, namely the *Camelus* and Hippotraginae lineages, as the foreground lineages, while other non-arid-adaptive species were defined as the background. And then, we used RELAX in Hyphy^29,109^ to test the gene-wide evolved pattern in arid-desert-adapted lineages by inferring a relaxation parameter K. We classified the patterns of selection pressure acting on genes. If a gene meets the criterion *dN/dS*_*foreground*_ > *dN*/*dS*_*background*_ and *K* > 1 (Likelihood Ratio Test, LRT, Benjamini–Hochberg, adjust *p*-value < 0.05), it is considered to be under intensified positive selection. Conversely, if a gene meets *dN/dS*_*foreground*_ > *dN*/*dS*_*background*_ but *K* < 1 (Likelihood Ratio Test, LRT, Benjamini–Hochberg, adjust *p*-value < 0.05), it is under relaxed positive selection. For intensified purifying selection, a gene must meet *dN/dS*_*foreground*_ < *dN*/*dS*_*background*_ and *K* > 1 (Likelihood Ratio Test, LRT, Benjamini-Hochberg, adjust *p*-value < 0.05). Lastly, if a gene satisfies *dN/dS*_*foreground*_ <*dN*/*dS*_*background*_ and *K* < 1 (Likelihood Ratio Test, LRT, Benjamini–Hochberg, adjust *P*-value < 0.05), it is under relaxed purifying selection. Our results showed that intensified positive selection played prominent roles in arid-desert adaptation. Convergent positive selection genes (CPSG) were identified by BUSTED-PH module and correcting for multiple testing (adjusted *P*-value (background) > 0.05, adjusted *P*-value (foreground) < 0.05, adjusted *P*-value (difference between background and foreground) < 0.05), Benjamini–Hochberg. Gene Ontology (GO) enrichment analysis was conducted by KOBAS (Xie et al. 2011).

We also employ the Mixed Effects Model of Evolution (MEME) (Murrell et al. 2012) in hyphy to test whether PAAS is under positive selection pressure. For each site, MEME infers two ω values and the probability of evolving under these ω values for a given branch. To infer ω, MEME calculates α (*dS*) and two distinct β (*dN*), namely β− and β+. A site is inferred to be under positive selection pressure when β+ > α and the likelihood ratio test indicates *p* < 0.05.

### Detection of parallel amino acid substitutions

At the beginning of identifying parallel amino acids substitution (PAAS) shared between arid-desert-adapted lineages, we inferred the ancestral sequence through Codeml program PAML (V4.9)^107^ in the internal nodes of 22 species phylogenetic tree, and then performed paired convergence analysis during *Camelinae* and Hippotraginae lineages, which includes three species and two ancestral nodes, respectively. In addition, to make a reliable signal of convergent molecular evolution at the amino acid level, four methods were conducted to try to find reliable PAAS. As the first measure, Profile Change with One Change (PCOC) pipeline^34^, can detect not only the same parallel substitutions of amino acids sites but also convergent shifts with similar biochemical properties that correspond to phenotypically convergent clades. We employed the PCOC to get a relaxed set of convergent genes in 25 pairwise combinations of two arid-desert-adapted linages, and the posterior probabilities (PP) for the PCOC model were set to greater than 0.99. And then, we expand CCS^73^, a strict method was applied in order to filter out noises as much as possible. In our study, considering that there may be more than one amino acid substitution at a particular site that contributes to the convergent phenotype under some circumstances, so moderate conservative sites follow three characteristics in foreground lineages: from different ancestral state to specific derived state, or from specific ancestral state to specific derived state, or from specific ancestral state to different/any derived state (Supplementary Fig. 3). Seven control groups were analyzed using the same procedure to obtain the corresponding sites and their gene sets. In developing the approach of Convergence Event Counting and Probability Calculation (Conv_cal)^38^, molecular convergence was considered to include both parallel substitutions which means identical substitutions in the target clades derived from the same ancestral amino acids and convergent substitutions which means the same substitutions changed from different ancestral amino acids. We herein collectively refer to these substitutions in all methods as parallel amino acid substitutions. To further exclude noise resulting from random amino acid substitutions, we put the JTT-fsite model of the Conv_cal method to figure out the observed number of convergence events through protein sequence alignments, and calculate the expected total probability of parallel and convergent events accordingly. Moreover, the Poisson test was employed to eliminate the noise generated by random amino acid substitution, and then applied the Benjamini–Hochberg (BH) method to correct the *P*-values, setting the false discovery rate (FDR) threshold at 0.1, to eliminate sampling bias between the experimental and control groups. Finally, CSUBST^40^ were used to detect adaptive substitutions that occur at the same protein site in multiple independent branches by calculating ratio between non-synonymous and synonymous substitution rates. We retained candidates with ωc > 1 in any catalog among convergence: omegaCany2spe and omegaCspe2spe, discordant convergence: omegaCdif2spe, and other profile convergence: omegaCspe2dif and omegaCspe2any. “csubst site” command can produce site-wise convergence/divergence probabilities with bar charts.

### Protein 3D structure simulation

We used I-TASSR^110–112^ to generate a homology model of the proteins (INSIG1 and NPC1L1) based on the human protein. Alphafold2^113^ was used to conduct three-dimensional (3D) structure simulations to examine the possible effects of these mutations. Homology modeling was used the protein sequences of the human in the Alphafold2. Structure visualization and manipulation were done in UCSF Chimera^114^. ProtScale (https://web.expasy.org/protscale/) were used to predict the hydrophobicity changes of INSIG1 and NPC1L1 between wild-type and mutant amino acids. FoldX^115^ was used to calculate the impact of point mutations on free energy, thereby assessing the effects on protein stability, folding, and binding.

### Metabolome analysis

Animals were fasted for 12 h (food withdrawn, water ad libitum). Housing was adjusted to prevent access to chow during fasting (timed removal, single-housing if necessary). Serum from camels and mice, fasted for twelve hours, was collected for off-target metabolomics analysis. Untargeted metabolomics was performed by Nuomi Metabolomics (Nanjing, China). The company provided the raw mass spectrometry files and the processed quantitative metabolite matrix; both were used for downstream analyses. Through peak detection, peak filtering and peak alignment processing, the material quantity is obtained, and the data is corrected by the area normalization method to eliminate systematic errors. For biochemical identification, Human Metabolome Database (HMDB)^116^ (http://www.hmdb.ca), massbank^117^ (http://www.lipidmaps.org), mzclound^118^ (https://www.mzcloud.org), KEGG^119^ (https://www.genome.jp/kegg/) and Pano Mick’s self-built standard product database (https://www.panomix.com/) were used. These characteristics include retention time, molecular weight to charge ratio (m/z), and associated chromatographic data (including MS/MS spectra). We ultimately identified 168 and 318 metabolites from the serum metabolomes of camels and mice, respectively, with four biological replicates. Enrichment analysis was conducted in MetaboAnalyst 5.0^120^ using the compound IDs to map to the 35 super chemical class metabolite sets based on chemcial structures. Differential metabolites between *Insig1*^*H132R/H132R*^ edited mice and wild-type mice were screened based on the thresholds of VIP > 1, *p*-value < 0.05, and abs(log2FoldChange) > 1.

### Cell culture and transfection

HepG2 cells (Qingqi, China) were cultured in DMEM medium (Bio-Channel, China) supplemented with 10% fetal bovine serum (TIANHANG, China) and 1% penicillin-streptomycin. HepG2 cells were cultured in a constant-temperature cell culture incubator set at 37 °C with 5% CO_2_. Before transfection, HepG2 cells were seeded in 6-well plates at a density of 6 × 10^5^ cells/well, and when the cell density reached about 70%, the optimized vector was transfected into HepG2 cells with PEI 40 K Transfection Reagent (Servicebio, China). Cell lysates were collected at 24 h and 48 h post-transfection for subsequent RT-qPCR and co-immunoprecipitation (Co-IP) analyses.

### Co-immunoprecipitation

HepG2 cells were cultured in 6-well plates until reaching ~80–90% confluence. After removing the culture medium, cells were gently washed twice with 1× PBS. Each well was then lysed with 200 μL of Co-IP/WB Tissue/Cell Lysis Buffer containing protease inhibitors (Affinibody, AIWB-012) and incubated on ice for 15 min. Lysates were centrifuged at 12,000 × *g* for 5 min at 4 °C, and the supernatants were collected as total protein extracts.

Protein A/G Magnetic Beads (MedChemExpress, HY-K0202) were thoroughly resuspended prior to use. Fifty microliters of beads were washed three times with 400 μL of binding/wash buffer (PBST, pH 7.4). The washed beads were incubated with HA antibody (Abmart, TT0050; final concentration 50 μg/mL) or isotype control IgG (Abmart, B30011) at 4 °C for 2 h with gentle rotation (10–15 rpm) to form antibody–bead complexes. Following incubation, the beads were separated using a magnetic stand and washed four times with binding/wash buffer.

Prepared cell lysates were then added to the antibody–bead complexes and incubated at 4 °C for an additional 2 h with gentle rotation (10–15 rpm) to capture antigen–antibody complexes. Subsequently, beads were washed four times with binding/wash buffer to remove nonspecific proteins. Bound proteins were eluted with acidic elution buffer (0.15 M glycine, pH 2.8), and the eluates were immediately neutralized with 0.1 M NaOH (1/10 of the total volume). Co-Immunoprecipitation Antibody Information were provided in Supplementary Data 13.

### Western blot analysis

Eluted proteins were mixed with 5×SDS-PAGE loading buffer and denatured at 37 °C for 30 min. Equal volumes of samples were separated by SDS-PAGE and transferred onto PVDF membranes (Millipore, USA). The membranes were blocked with a dedicated protein blocking buffer (Servicebio, G2052-500ML) for 30 min at room temperature to minimize nonspecific binding. Subsequently, the membranes were incubated overnight at 4 °C with primary antibodies diluted 1:1000 in TBST containing 5% BSA. The primary antibodies used were anti-HA (Abmart, T62939) and anti-FLAG (Abcepta, AP74805).

After washing three times with TBST, the membranes were incubated with HRP-conjugated goat anti-mouse IgG (H + L) secondary antibody (Proteintech, SA00001-1) at a 1:10,000 dilution for 2 h at room temperature. Protein signals were detected using an enhanced chemiluminescent (ECL) substrate (Affinibody, China) and visualized with a fluorescence/chemiluminescence imaging system (Peiqing, China).

### Dil-LDL uptake assay

The LDL-C uptake was determined using fluorescently labeled Dil-LDL (L3482, Thermo Fisher Scientific, MA, USA) in HepG2 cells according to the manufacturer’s protocol. Briefly, cells were cultured in serum-free medium for 24 h and then incubated in serum-free medium containing 5 mg·mL-1 Dil-LDL for 4 h in the dark. Then, cells were stained with DAPI for 20 min and washed 3 times with PBS. The images were obtained with a fluorescence microscope (Spinning Disk Confocal Microscope, Revolution WD, Andor, England). The Dil-LDL-stained cell membrane showed orange-red fluorescence, and the DAPI-stained cell nucleus showed blue fluorescence. For semiquantitative analysis, the staining intensity was measured using ImageJ software. The normalized values have been placed in the Supplementary Data 15.

### Oil Red O staining

For Oil Red O staining, cells were first washed three times with PBS, then fixed with 4% paraformaldehyde for 20–30 min, and washed again three times with PBS. After fixation, cells were incubated with freshly prepared Oil Red O working solution for 10–20 min. The dye was then removed, and cells were washed three times with PBS. Finally, PBS was added to uniformly cover the cells, and images were acquired under a light microscope. For semiquantitative analysis, the staining intensity was measured using ImageJ software. The normalized values have been placed in the Supplementary Data 14.

### Plasmid constructs

The expression plasmids used in this study were generated based on the pcDNA3.1(+) vector (Invitrogen, USA) using standard molecular cloning techniques. The coding sequences of human INSIG1 (GenBank accession No. NM_005542) and SCAP (GenBank accession No. NM_012235) were amplified and inserted into the pcDNA3.1(+) backbone. For INSIG1, a C-terminal HA tag was introduced to generate the HA-INSIG1(WT) construct, while the H150R point mutation (A449G substitution, (CAC → CGC)) was introduced by site-directed mutagenesis to produce HA-INSIG1(H150R). The SCAP construct was designed with a C-terminal FLAG tag to obtain FLAG-SCAP. All recombinant plasmids were confirmed by Sanger sequencing to ensure the accuracy of the open reading frame and the presence of the desired mutation. The plasmid information is summarized in Supplementary Data 24.

### cDNA preparation

Total RNA was reverse-transcribed into complementary DNA (cDNA) using the SmArt RTMaster Premix (5×) (Cat. No. DY10502; Deeyee, China) according to the manufacturer’s instructions. Each 20 μL reaction contained 4 μL of 5× SmArt RTMaster Premix, an appropriate amount of total RNA (10 ng–2 μg), and RNase-free water to reach a final volume of 20 μL. The premix included SmArt RTase (a thermostable M-MLV reverse transcriptase lacking RNase H activity), RNase inhibitor, random primers, oligo(dT) primers, dNTP mixture, and an optimized reaction buffer containing Mg2+. The reverse transcription reaction was performed under the following thermal conditions: 25 °C for 5 min, 50 °C for 15 min for reverse transcription, and 85 °C for 5 s to inactivate the enzyme, followed by holding at 4 °C. The resulting cDNA was used as the template for subsequent real-time quantitative PCR (RT-qPCR).

### Real-time quantitative PCR

Real-time quantitative PCR (qPCR) was performed using the CFX96 Real-Time PCR Detection System (Bio-Rad, Hercules, CA, USA) with SYBR Green chemistry^121^. Each 20 μL reaction contained 10 μL of 2× Universal Blue SYBR Green qPCR Master Mix (G3326-05, Servicebio), 0.4 μL of forward primer (10 μM), 0.4 μL of reverse primer (10 μM; final concentration 0.2 μM each), 1 μL of cDNA template diluted 1:10 from reverse transcription, and nuclease-free water to volume. The thermal cycling program was as follows: initial denaturation at 95 °C for 3 min, followed by 40 cycles of 95 °C for 10 s and 60 °C for 30 s, during which fluorescence signals were collected. Melting curve analysis was performed using a stepwise protocol: 95 °C for 15 s, 60 °C for 1 min, and then an incremental increase from 60 °C to 95 °C at 0.3 °C per step with a 15 s hold at each step for signal acquisition. Amplification specificity was confirmed by the presence of a single melting peak. All reactions were conducted with at least three technical replicates per sample. two-tailed Student’s *t* test. The sequence of primers used for qPCR is listed in Supplementary Data 19.

### Generation of *Insig1*^*H132R/H132R*^ mutation mice

For in vitro assays in HepG2 (human), we used the human INSIG1(H150R) construct following human residue numbering; for the knock-in mouse line we edited the orthologous mouse residue Insig1(H132R), as determined by human–mouse INSIG1/Insig1 sequence alignment.

CRISPR-Cas9-mediated genome editing was employed to generate mice harboring the Insig1(H132R) point mutation at Cyagen (Suzhou) Biotechnology Co. Ltd. (Jiangsu, P. R. China). A CRISPR/Cas9 knock-in strategy was used to introduce the Insig1 p.H132R missense substitution (CAC → CGC). Cas9, a single guide RNA (sgRNA, gRNA-A1: 5′-TCCCTGTATTGACAGTCACCTGG-3′, matching the forward strand of Insig1), and a single-stranded donor oligonucleotide (ssODN) were co-injected into fertilized mouse zygotes. The ssODN sequence was: 5′-TCCTGTTTTGTTTTTCTTTAAACAGCTGTTGTCGGTTTACTGTATCCCTGTATTGACAGTCGCCTGGGAGAACCACACAAGTTCAAGAGAGAATGGGCCAGCGTTATGCGCTGTATTGCCGTG-3′ (where the wild-type codon CAC was replaced by CGC to generate the p.H132R substitution). Founder (F0) animals were identified by PCR amplification of the target locus followed by Sanger sequencing. A total of four positive F0 mice were obtained; among these, the F1 animals delivered to the client colony all derived from a single positive male founder (F0-6).

### Genotyping and sequence validation

PCR genotyping was performed with primers F1 (forward, 5′-TGCCGAGGAAAATAAGTGGTTTGG-3′) and R1 (reverse, 5′-AGCCACAGTAAACCTCTGCTTCTA-3′), using LongAmp Taq DNA polymerase (NEB M0323L) for 33 cycles at an annealing temperature of 60 °C, which yielded a 403-bp amplicon. A no-template control (NTC; water) and a wild-type genomic DNA control were included in each PCR run to monitor contamination and verify assay performance. PCR products were verified by Sanger sequencing using F1 as the sequencing primer. Representative allele sequences at the edited motif were: WT …TATTGACAGTCACCTGGGAGA… and mutant …TATTGACAGTCGCCTGGGAGA…. Tail DNA was prepared either with a silica-column kit (TaKaRa MiniBEST, code 9765) or a proteinase-K lysis buffer (50 mM KCl, 10 mM Tris-HCl pH 9.0, 0.1% Triton X-100, 0.4 mg mL⁻¹ proteinase K).

### Colony establishment

Positive founder F0-6 (♂) was bred to WT C57BL/6J to assess germline transmission and establish F1 animals. The cross yielded six F1 offspring (3 males, 3 females; DOB 2023-01-15), each carrying the p.H132R allele by PCR and Sanger sequencing. Heterozygous F1 mice were intercrossed to generate F2 litters; genotyping and sequencing (as above) were used to assign *Insig1*^*H132R/H132R*^, Insig1^+/H132R^ and Insig1^+/+^ genotypes.

### Experimental cohorts and background strain

Experiments used male *Insig1*^*H132R/H132R*^ mice and WT littermates aged ≥3 months. We did not take off-target effects into account. The line was maintained on a C57BL/6JCya background. For reference, C57BL/6JCya identifiers are MGI: 7786639.

### Randomization

Edited and wild-type mice were housed at the Animal Core Facility of Northwest A&F University under identical husbandry conditions. Cage assignments and processing order were determined according to a randomization schedule, with allocation performed by a researcher who did not take part in outcome assessment. To minimise potential confounding, measurements and sample processing were performed in balanced batches, and the order of animals within each batch was randomized.

### Breeding, husbandry and ethics

All experimental mice were bred and reared under standard conditions in the investigator’s colony at the Animal Core Facility of Northwest A&F University (Shannxi, P. R. China, certificate no. SCXK [SHAAN] 2017-003). Approval for all experimental procedures was obtained from Northwest A&F University.

### Transcriptomic analysis

For cross-species transcriptomic analysis, we downloaded publicly available transcriptome data comprising eight samples each of camels, cattle, and humans. Data underwent quality control using Trimmomatic^122^. Subsequently, clean fastq were mapped to their respective NCBI reference genomes, namely Ca_bactrianus_MBC_1.0, ARS-UCD1.2, and GRCh38.p14, using HISAT2^123^. Gene expression levels were quantified using StringTie^124^. We have provided detailed information in the attached Supplementary Data 12 regarding the publicly available data downloaded from NCBI, including Accession (PRJNA857334, PRJEB35350, PRJNA844027, PRJNA665193, PRJNA522422, PRJNA518006), Run, Sample, Species, Tissue, Years, Sex, and Condition. The last two columns in Supplementary Data 12 represent the FPKM values for gene-INSIG1 and gene-NPC1L1.

Liver and epididymal white adipose tissue (eWAT) from three genome-edited and three wild-type mice were submitted to Novogene (Beijing, China) for library preparation and sequencing^125^. We received the raw FASTQ files. Reads were quality-filtered with Trimmomatic, aligned to GRCm39 using HISAT2, and gene expression was quantified with StringTie. The reference genome version for mice was GRCm39. And then we calculated differentially expressed genes (DEGs) using DESeq2^126^ with threshold of adjust *p*-value < 0.05 and abs(log2FoldChange) > 1. Gene Set Enrichment Analysis (GSEA)^67^ were used to interpreted gene expression data between gene-edited and wild-type mice liver and visceral fat. KEGG pathways and GO terms were enriched by KOBAS^127^ with threshold of adjust *p*-value 0.05 based DEGs from gene-edited and wild-type mice liver and visceral fat.

### Statistics and reproducibility

All experiments were repeated at least three times, and representative data are presented. For Dil-LDL uptake assay, we conducted 4 biologically independent experiments. For Oil Red O staining, 5 biologically independent experiments were conducted. One-way ANOVA with Tukey’s post-hoc test were used.

For metabolomics, blood samples from both camels (*n* = 4 male) and mice (*n* = 4 male) were collected using non-invasive methods. Differential metabolites between *Insig1*^*H132R/H132R*^ edited mice and wild-type mice were screened based on the thresholds of VIP > 1, *p*-value < 0.05, and abs(log2FoldChange) > 1.

For transcriptomics, liver and epididymal white adipose tissue (eWAT) from 3 genome-edited and 3 wild-type male mice. DEGs identified by threshold of adjust *p*-value < 0.05 and abs(log2FoldChange) > 1.

### Reporting summary

Further information on research design is available in the Nature Portfolio Reporting Summary linked to this article.

## Supplementary information

Supplementary Materials

Description of Additional Supplementary Files

Supplementary Data 1-24

Reporting Summary

## Data Availability

The genome data generated in this study have been submitted to the NCBI BioProject database (https://www.ncbi.nlm.nih.gov/bioproject/) under accession number: BioProject ID: PRJNA1158569; BioSample ID: SAMN43543267. Submitted GenBank assembly GCA_048773025.1 in this study has now been listed on the NCBI website as NCBI RefSeq assembly GCF_048773025.1 Addgene ID: 250155, 250158, 250159 The transcriptome data of mice has also been uploaded to NCBI with accession number: SRP620610. Detailed information can be found in the Supplementary Data 23. Numerical source data for graphs and charts can be found in Supplementary Data 4–23. The data that support the findings of this study are available from the corresponding author upon reasonable request.

## Code availability

The in-house Perl scripts have been uploaded to github (https://github.com/1221li/comparative-genome.git).
