# Supplementary material for: INSIG1 parallel substitution drives lipid/sterol metabolic plasticity mediating desert adaptation in ungulates
Source: Commun Biol. 2026 Jan 12;9:245. doi: 10.1038/s42003-026-09523-z (PMC12905343; doi:10.1038/s42003-026-09523-z)
Supplement: Supplementary file 1 — Supplementary Materials [file 42003_2026_9523_MOESM1_ESM.pdf]

Supplementary Materials for

INSIG1 parallel substitution drives lipid/sterol metabolic plasticity mediating desert adaptation in ungulates

Xinmei Li[1†], Ziyi He [1†], Anguo Liu[1], Fanxin Meng[1], Xiao Zhang[1], Nana Li [1], Huan Liu[1], Yuyi Lu[1], Zhipei Wu[1], Huimei Fan[1], Xixi Yan[1], Nange Ma[1], Zhenyu Wei[1], Wei Wang[1], Xixi He[1], Kunyu Ma[1], Yu Jiang[1], Chao Tong[3*], Bo Xia[1*], Yu Wang[1,2*]

*Corresponding author. E-mail: wang_yu@nwsuaf.edu.cn; imed23@nwafu.edu.cn; tongchao1990@gmail.com

This PDF file includes:

Figs. S1 to S15#

Other Supplementary Materials for this manuscript include the following:

Supplementary Data 1-24#

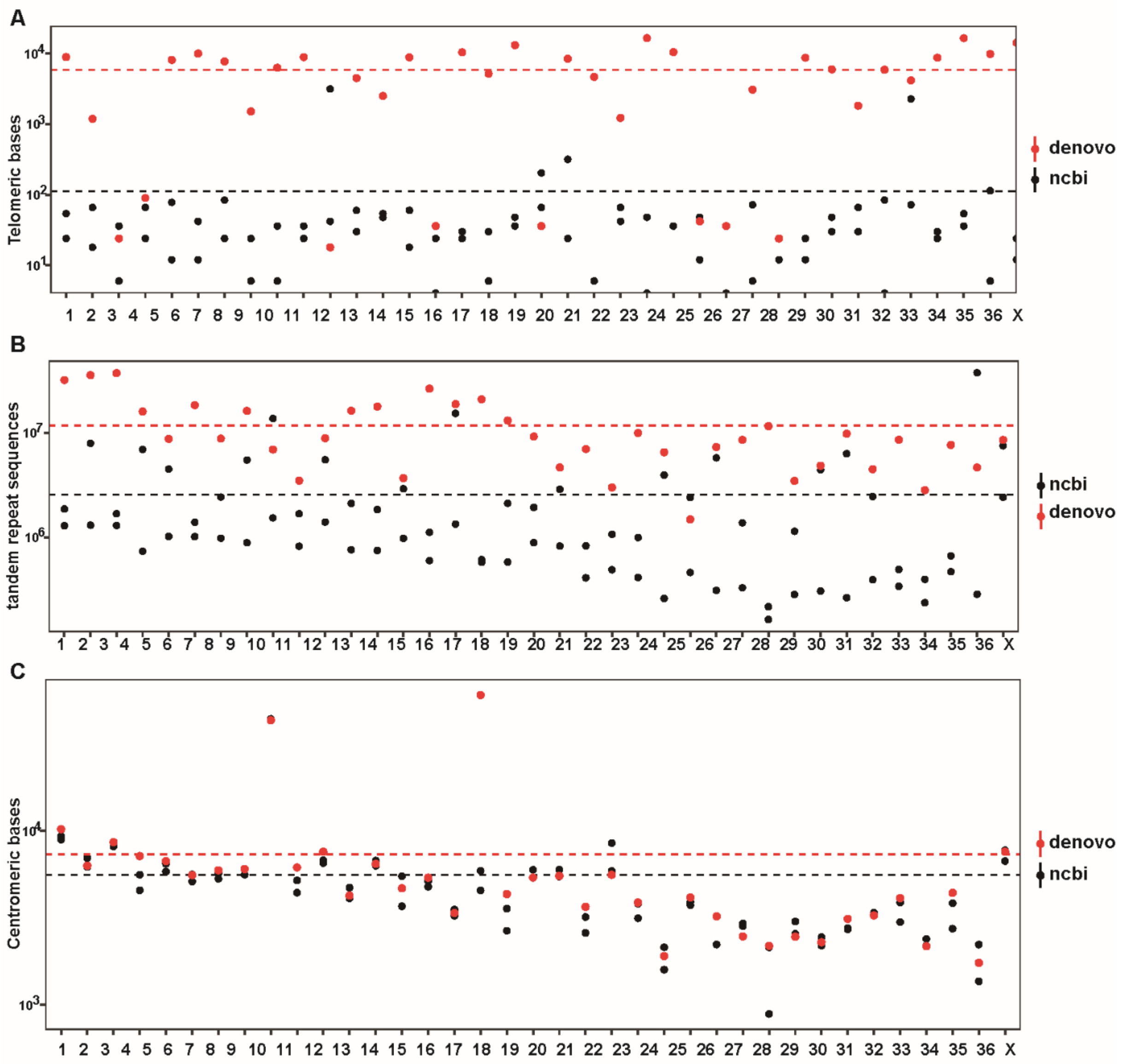

Supplementary Figure 1 The completeness of telomere and tandem repeat sequence.

(A) Statistical distribution of centromeric bases on each chromosome of *de novo* assembly (red dot) and ncbi reference genome of dromedary and wild Bactrian camel (black dots). The number of bases in telomeric repeats within 10 Kb of chromosome ends. Dashed lines indicate the autosome-wide mean for the respective color of points. (B) Tandem repeat sequences on each chromosome were identified by Tandem repeats finder (TRF).

(C) The mean number of bases on each chromosome identified as “Satellite” by RepeatMasker for the *de novo* (red) assembly and ncbi reference genome of dromedary and wild Bactrian camel (black). Dashed lines indicate the autosome-wide mean.

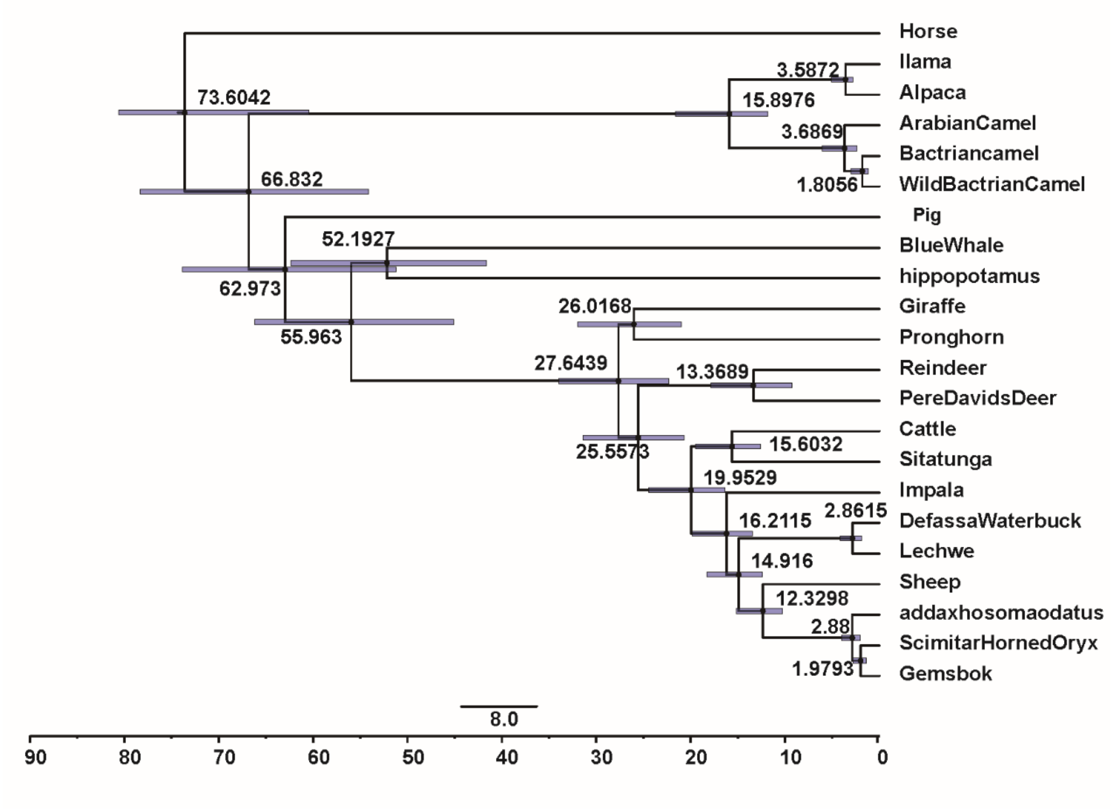

Supplementary Figure 2 Estimated divergence times among 22 ungulates.

The blue rectangles at the nodes represent 95% confidence intervals of the corresponding estimated divergence times.

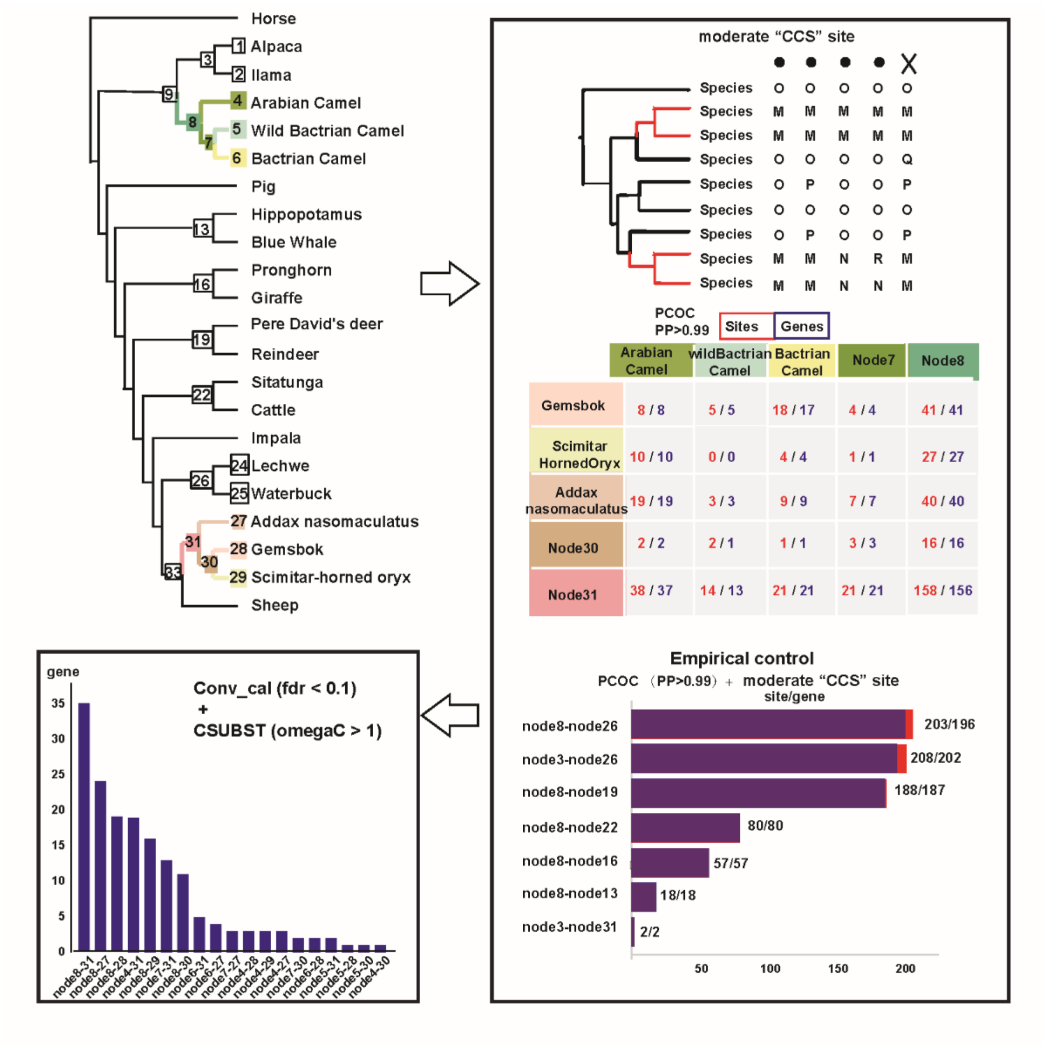

Supplementary Figure 3 Strategies for screening nonrandom candidate convergent gene sets.

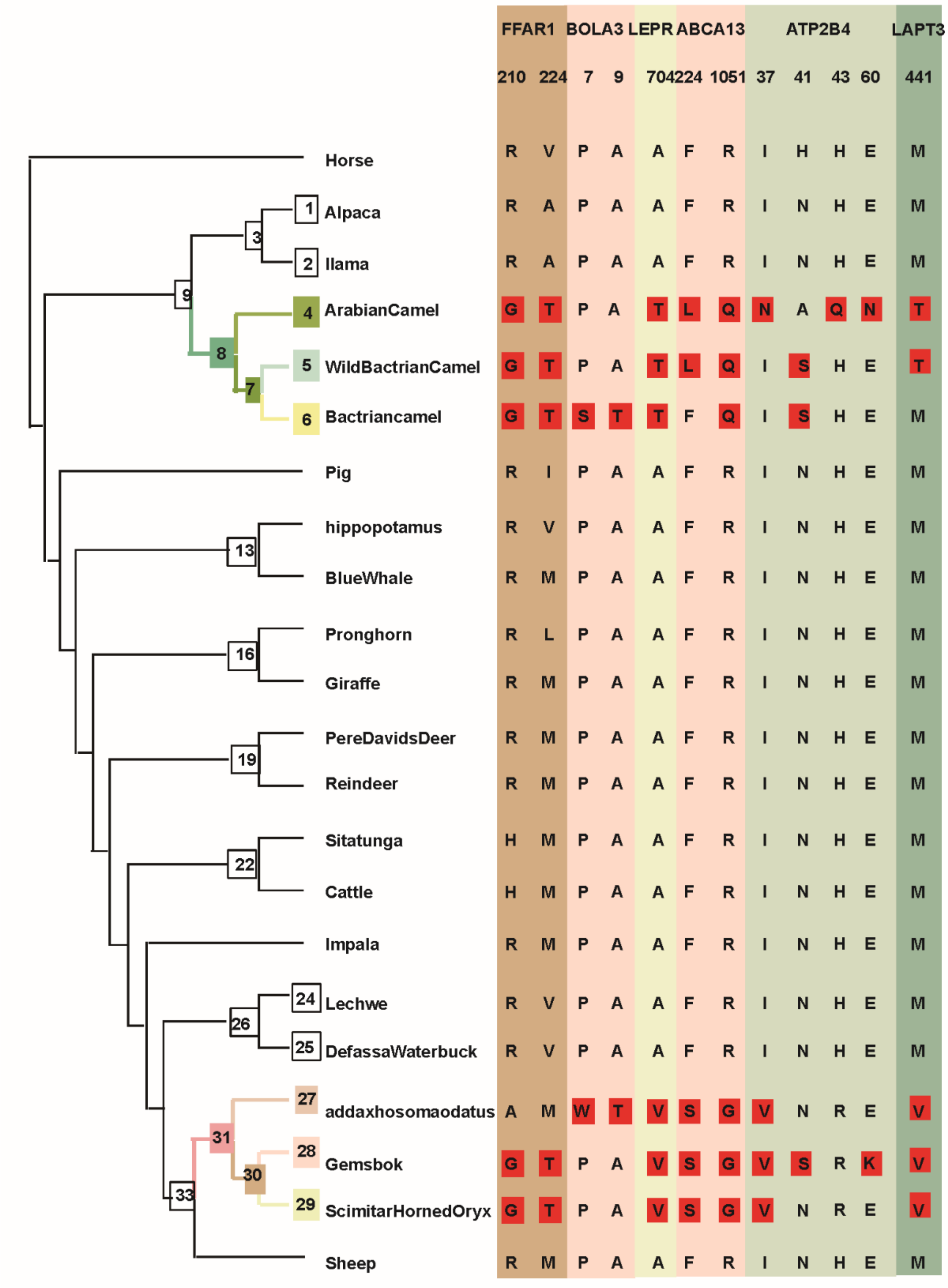

Supplementary Figure 4 Convergent genes FFAR1, BOLA3, ABCA13, ATP2B4, LAPT3.

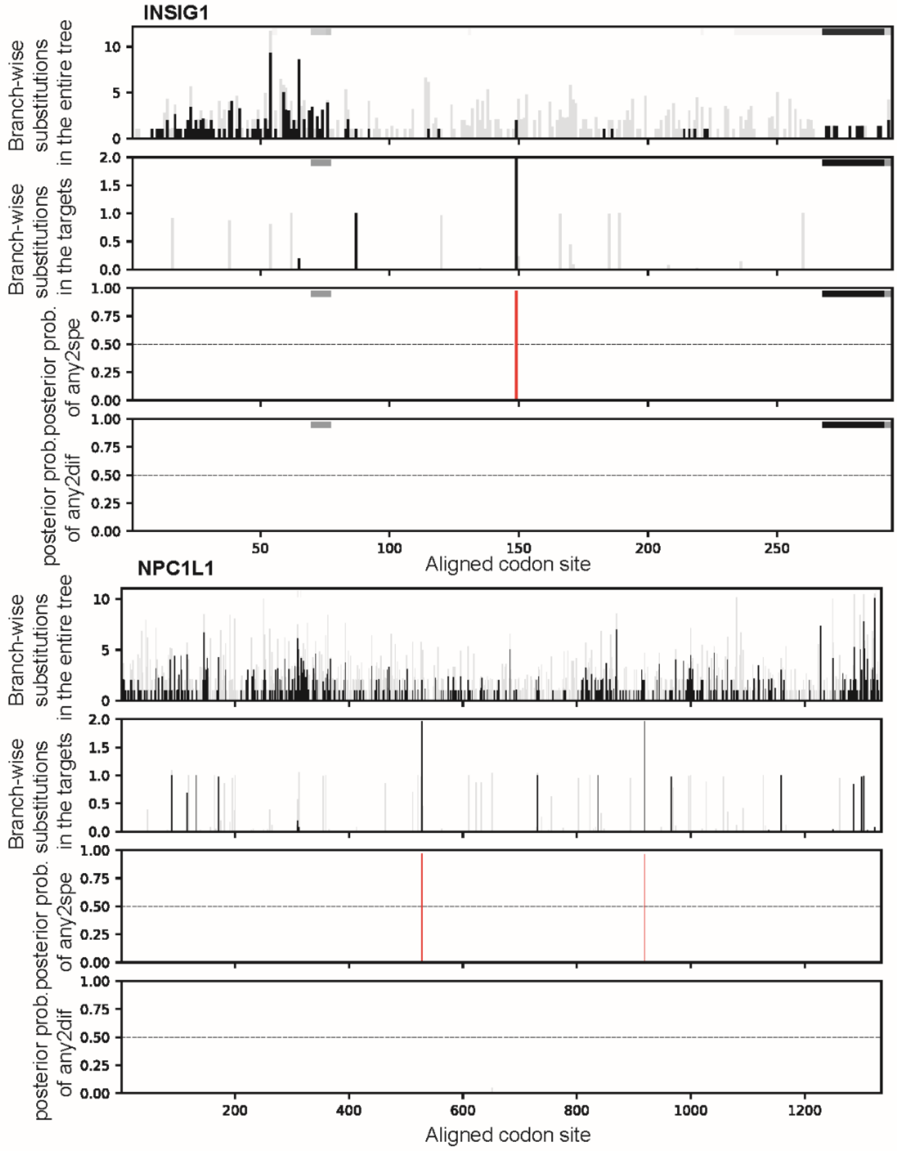

Supplementary Figure 5 The probability of substitution for each codon site of INSIG1 and NPC1L1 calculated by CSUBST. Black and gray represents nonsynonymous and synonymous substitutions, respectively. And red/blue is non-synonymous combinatorial substitutions.

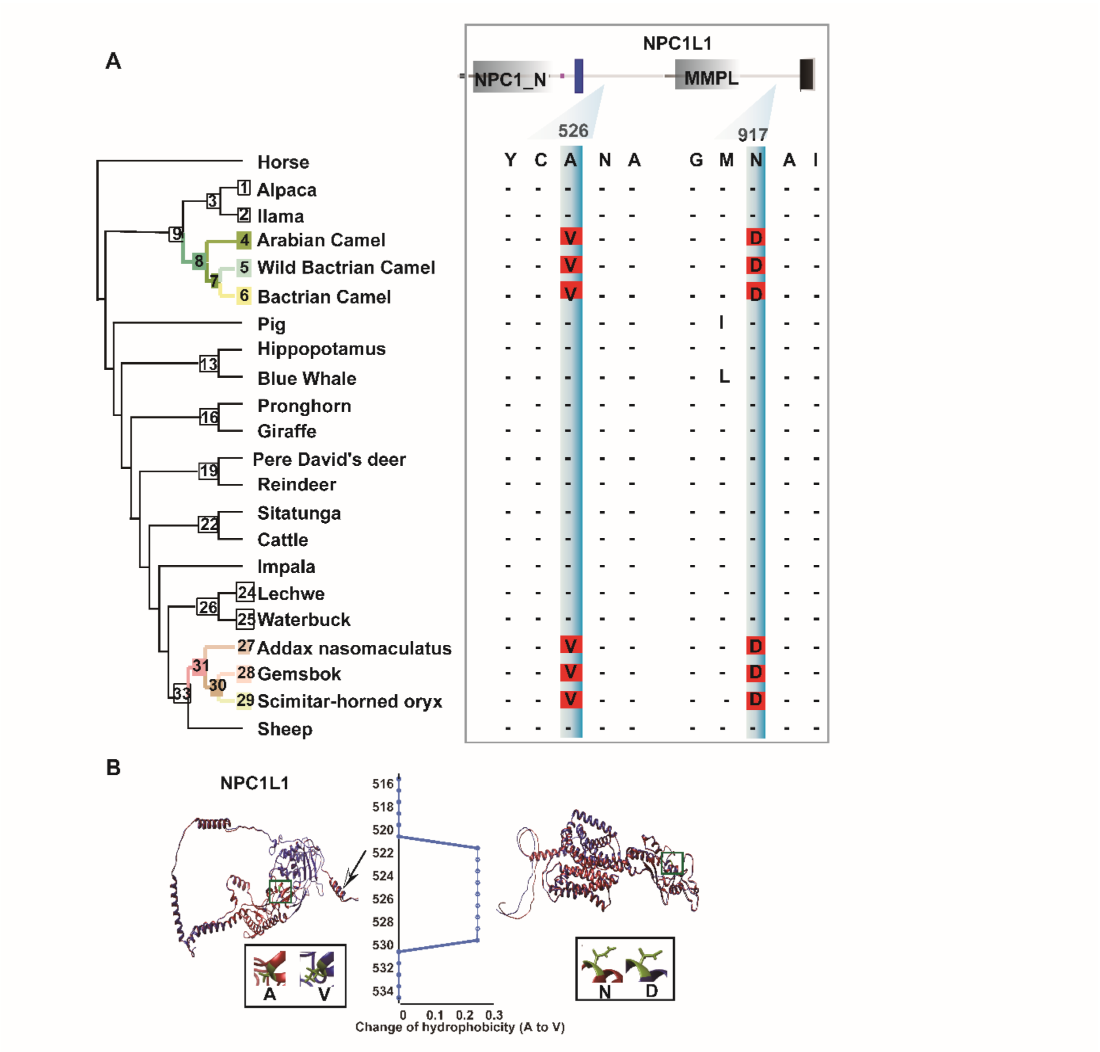

Supplementary Figure 6 adaptive convergent mutation in NPC1L1

(A) Parallel substitutions at conserved sites of NPC1L1 protein in Camelus and Hippotraginae lineages.

(B) Mutation site in the 3D protein structures of NPC1L1 was indicated in green. The red chains represent the normal protein structure, and the blue chains represent protein structure after parallel mutations. The arrows indicate the locations where structural changes occurred before and after the mutation. The hydrophobicity prediction of NPC1L1. Amino acid substitution of A526V in NPC1L1 increase the amino acids hydrophobicity from 522 to 530.

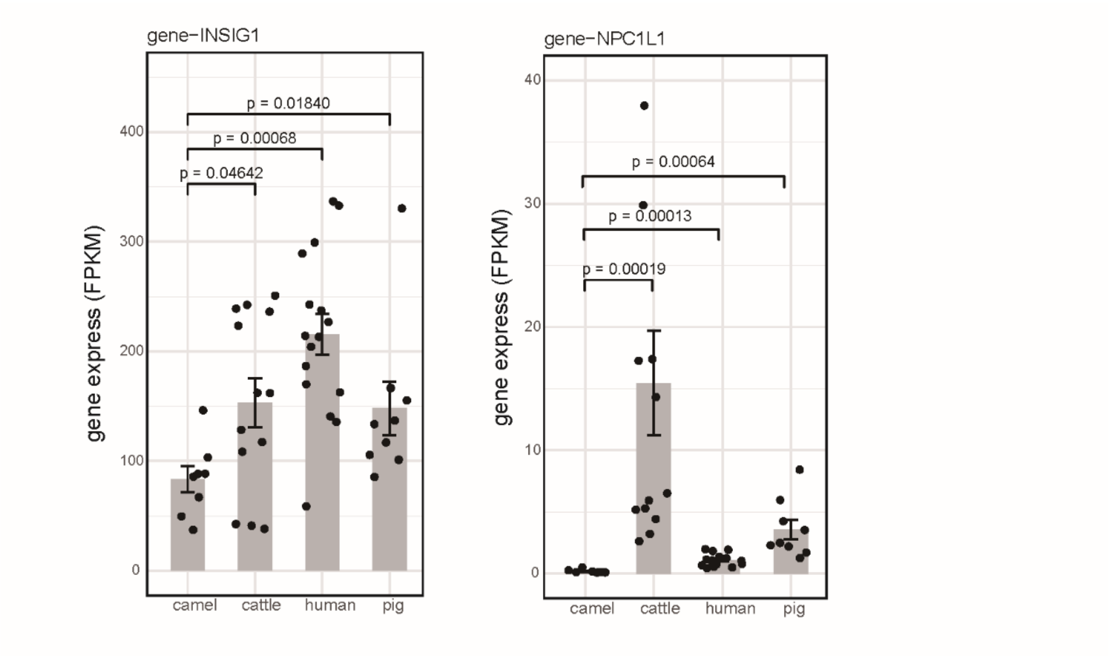

Supplementary Figure 7 Expression level of *NPC1L1* (left) and *INSIG1* (right) in camel, cattle, human, and pig. Statistical significance was assessed using rank sum test; *p ≤ 0.05, **p ≤ 0.01, ***p ≤ 0.001, ns = not significant. Values and error bars represented means and ± SEM from multiple independent biological replicates

WT

```
Query  .........10.........20.........30.........40.........50.........60.........70.........80.........90
A      MPRLHDHFWS CSCAHSARRR GPPRASAAGL AAKVGEMINV SVSGPSLLAA HGAPDADPAP RGRSAAMSGP EPGSPYPNTW HHRLLQRSLV
       .........100.........110.........120.........130.........140.........150.........160.........170....
       LFSVGVVLAL VLNLLQIQRN VTLFPEEVIA TIFSSAWWVP PCCGTAAAVV GLLYPCIDSH LGEPHKFKRE WASVMRCIAV FVGI
       .....180.........190.........200.........210.........220.........230.........240.........250........
       NHASAK LDFANNVQLS LTLAALSLGL WWTFDRSRSG LGLGITIAFL ATLITQFLVY NGVYQYTSPD FLYIRSWLPC IFFSGGVT
       .260.........270.........280.........290.........300.........310.........320.........330
       VG NIGRQLAMLI PFCEELNLKT TWLFHKTRSN YRVFLKSPIV IESSKPPILR ARKILEENLT VDYDKDYLFS
```

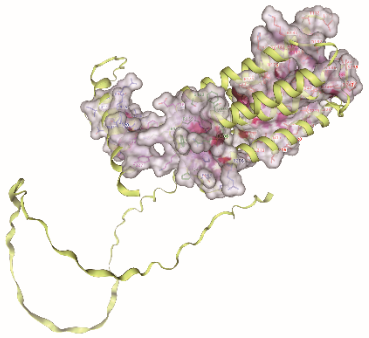

CurPocket ID	Cavity volume	Center (x, y, z)	Cavity size (x, y, z)
C1	4347	-6, 6, 6	30, 18, 27
C2	549	0, -29, -19	14, 11, 15
C3	319	8, -18, -10	12, 7, 10
C4	208	11, -13, 0	8, 9, 11
C5	134	17, -5, -3	5, 9, 5

H-R

```
Query  .........10.........20.........30.........40.........50.........60.........70.........80.........90
A      MPRLHDHFWS CSCAHSARRR GPPRASAAGL AAKVGEMINV SVSGPSLLAA HGAPDADPAP RGRSAAMSGP EPGSPYPNTW HHRLLQRSLV
       .........100.........110.........120.........130.........140.........150.........160.........170....
       LFSVGVVLAL VLNLLQIQRN VTLFPEEVIA TIFSSAWWVP PCCGTAAAVV GLLYPCIDSR LGEPHKFKRE WASVMRCIAV FVGI
       .....180.........190.........200.........210.........220.........230.........240.........250........
       NHASAK LDFANNVQLS LTLAALSLGL WWTFDRSRSG LGLGITIAFL ATLITQFLVY NGVYQYTSPD FLYIRSWLPC IFFSGGVT
       .260.........270.........280.........290.........300.........310.........320.........330
       VG NIGRQLAMLI PFCEELNLKT TWLFHKTRSN YRVFLKSPIV IESSKPPILR ARKILEENLT VDYDKDYLFS
```

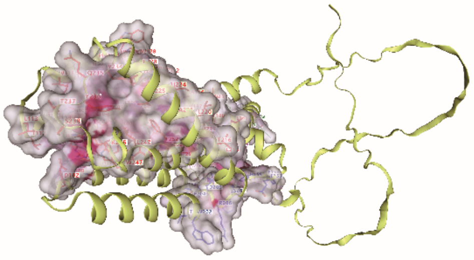

CurPocket ID	Cavity volume	Center (x, y, z)	Cavity size (x, y, z)
C1	3832	-6, 5, 6	30, 18, 27
C2	403	8, -15, -2	13, 9, 10
C3	261	5, -17, -12	12, 7, 7
C4	174	-5, -29, -29	9, 8, 11
C5	146	-15, -36, -41	7, 7, 6

Supplementary Figure 8 The curpocket and cavity for INSIG1(H150R) and wildtype INSIG1 identified by CB-Dock2.

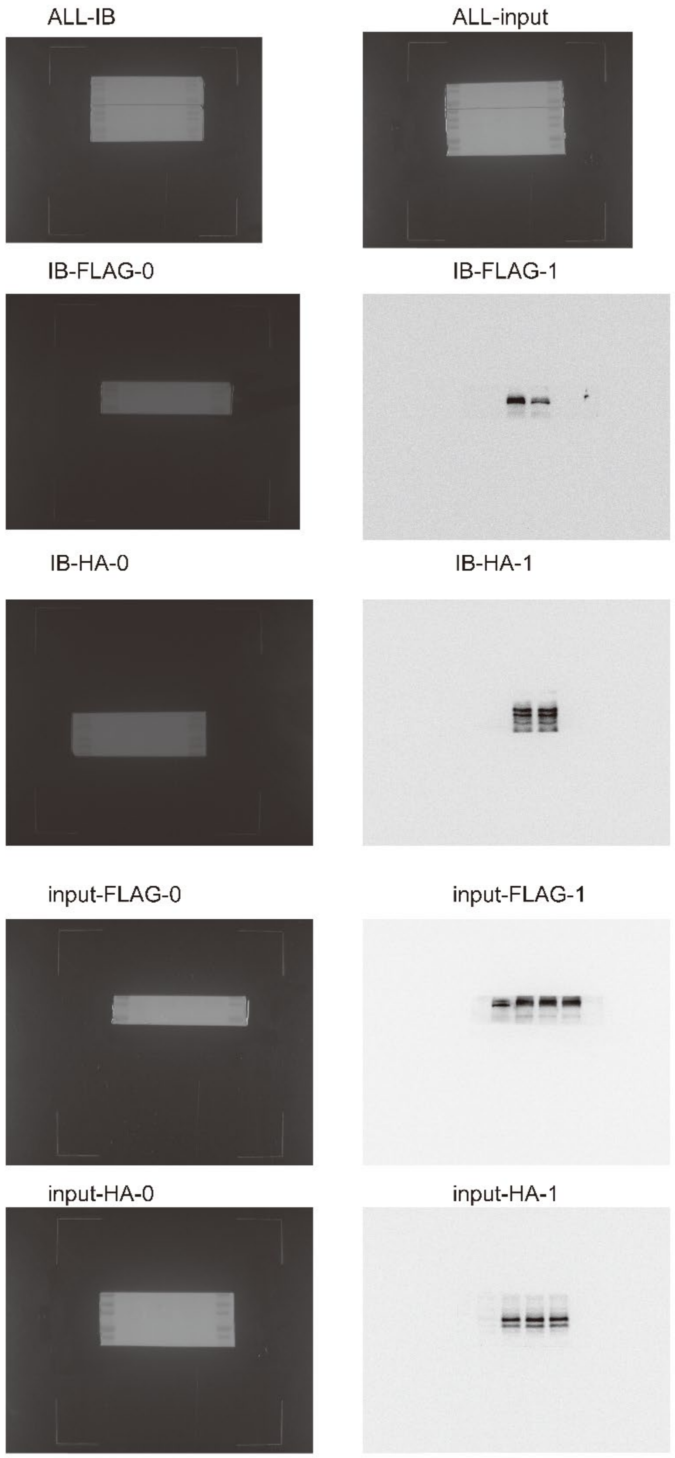

Supplementary Figure 9 Uncropped and unedited blot/gel images

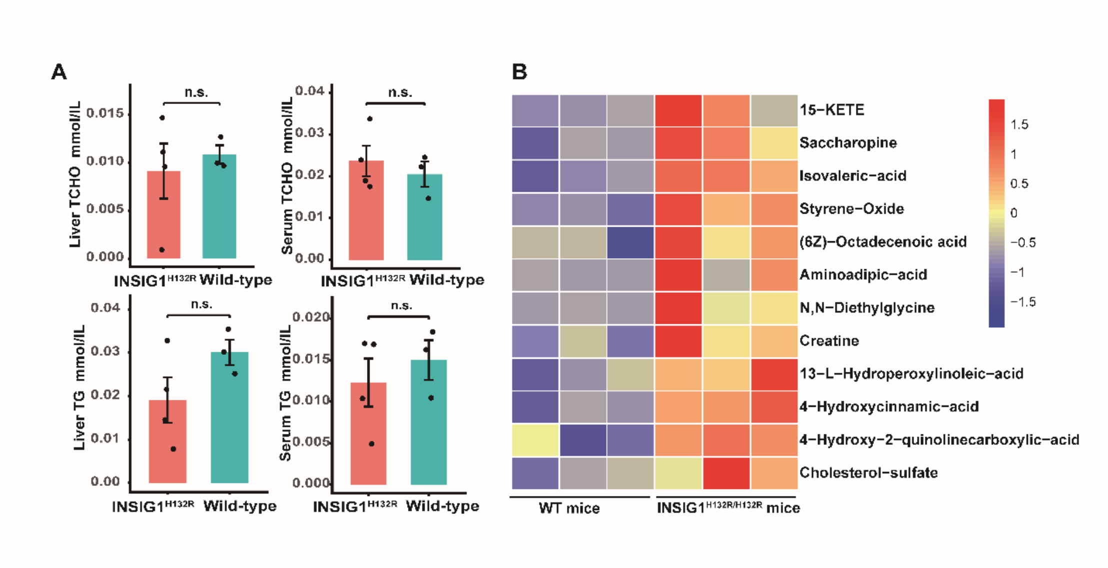

Supplementary Figure 10 The phenotypes of wild-type and *Insig1*$^{132R/132R}$ gene-edited mice

(A) The cholesterol (up) and triglyceride (down) concentrations in serum and liver of mutant (n=4 male mice) and wild-type mice (n=3 male mice). Welch Two Sample t-test, Values and error bars represented means and ± SEM from multiple independent biological replicates.

(B) Differential metabolites in the liver between mutant and wild mice.

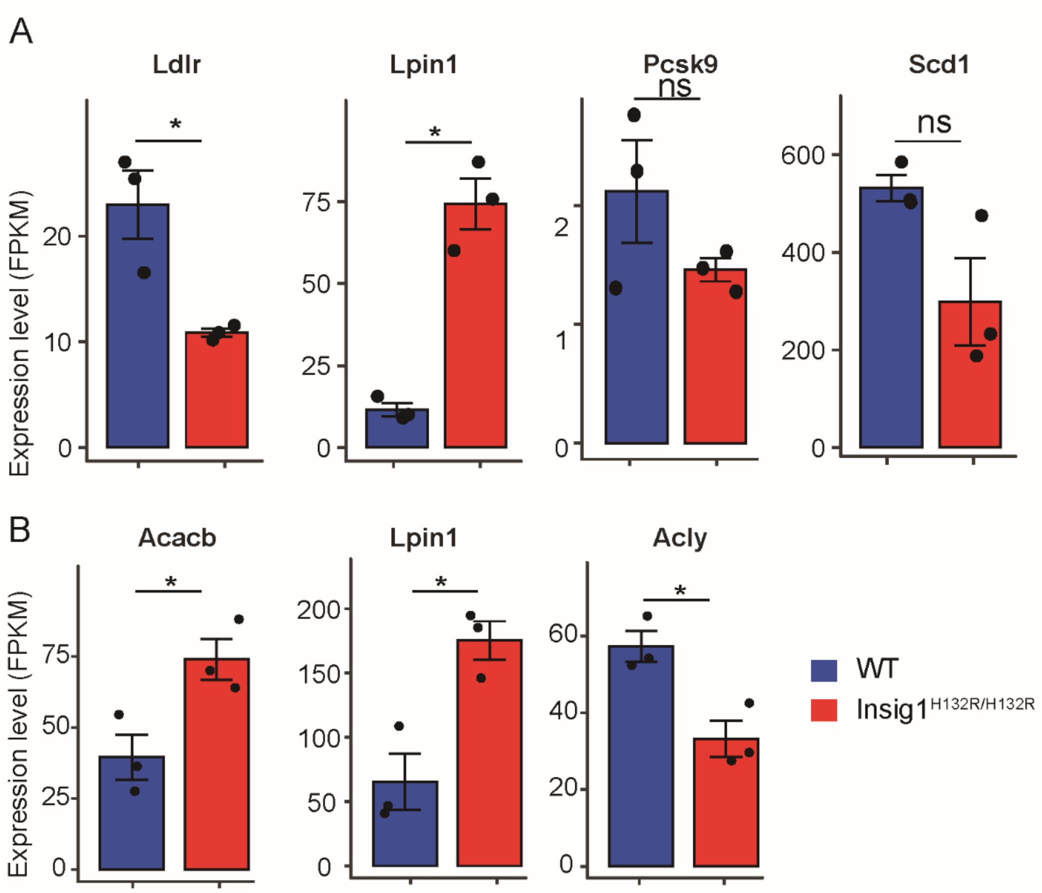

Supplementary Figure 11 The changes of SREBP target sites in the liver tissue (A) and epididymal white adipose (eWAT) (B) of *Insig1*$^{H132R/H132R}$ mice. *P≤0.05, **P≤0.01, ns: not significant by two-tailed Student's t test. Values and error bars represented means and ± SEM from multiple independent biological replicates

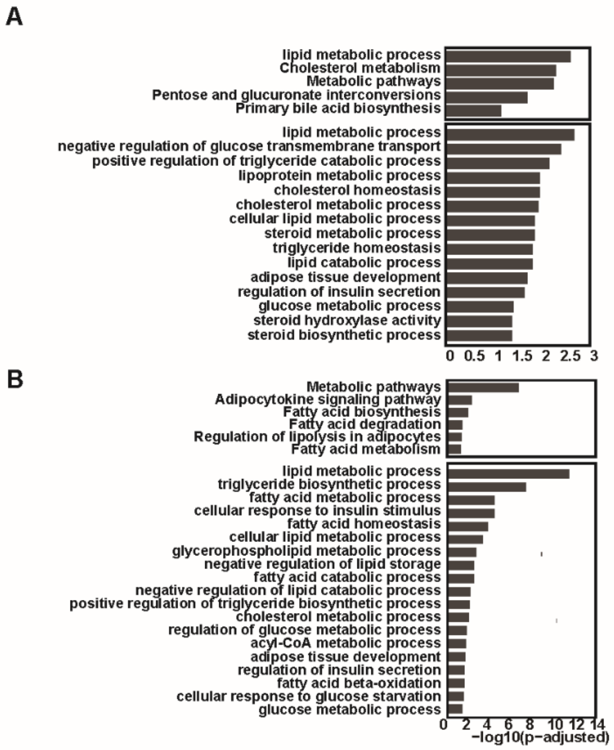

Supplementary Figure 12 The difference of transcriptome and metabolome between wild-type and gene-edited mice

(A-B) Pathways were enriched by differentially expressed genes in liver (A) and visceral fat (B) of homozygous and wild-type mice.

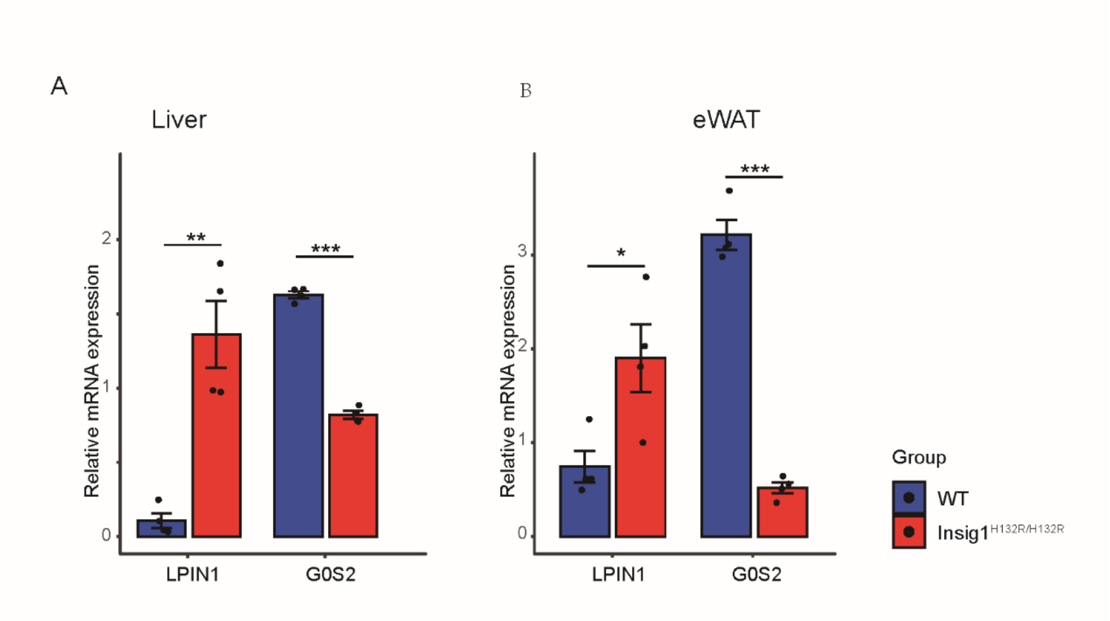

Supplementary Figure 13 DEGs in liver (A) and eWAT (B) verified by RT-qPCR. Statistical significance was assessed using two-tailed t-tests; *$p \leq 0.05$, **$p \leq 0.01$, ***$p \leq 0.001$, ns = not significant. Values and error bars represented means and ± SEM from multiple independent biological replicates

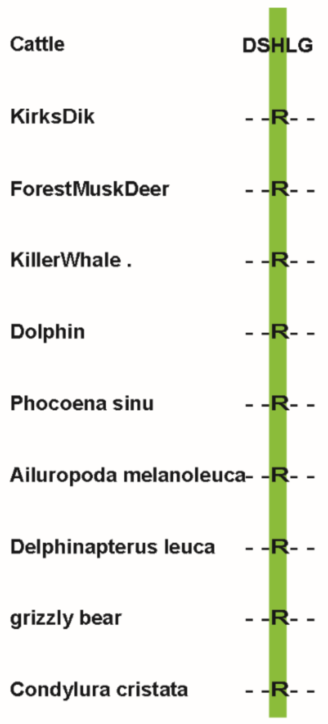

Supplementary Figure 14 the H-R mutation of INSIG1 in other more animals.

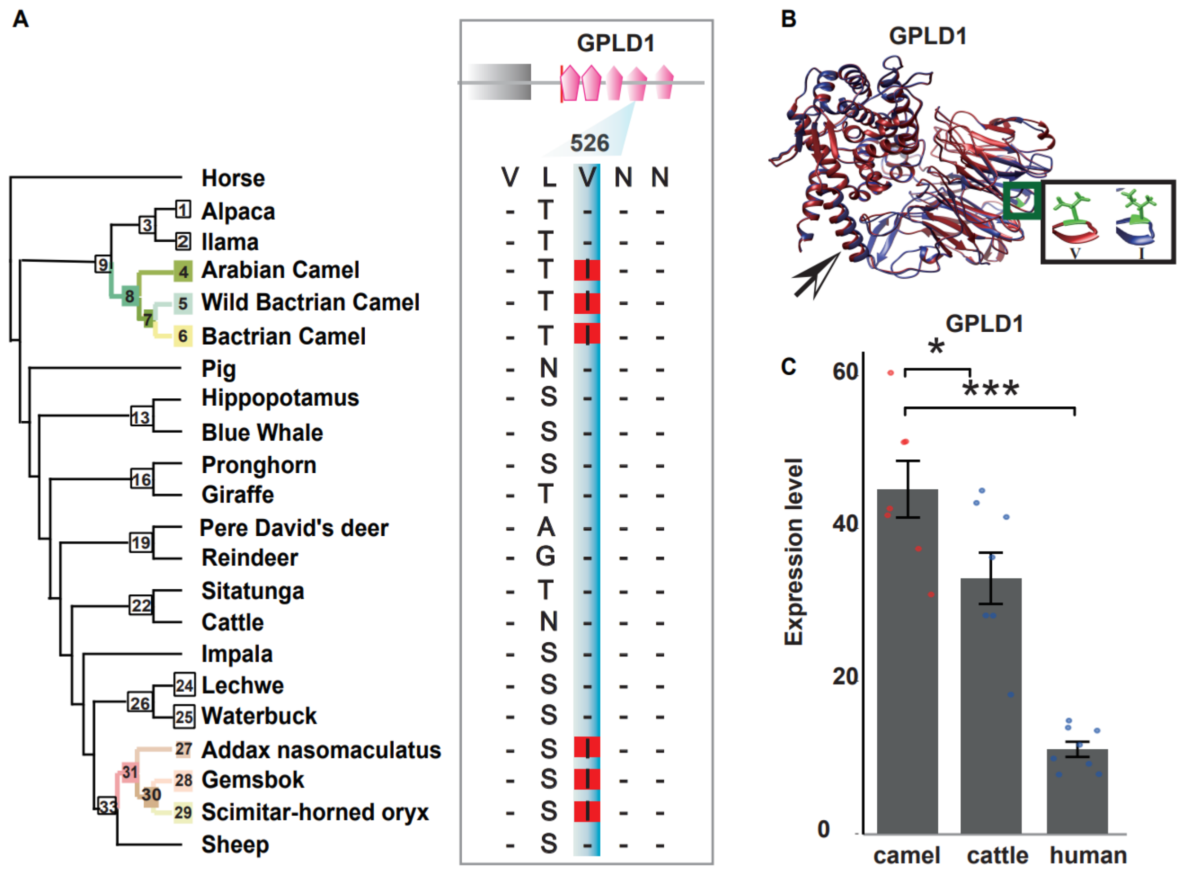

Supplementary Figure15 (A) Parallel substitutions at conserved site of GPLD1 protein in Camelus and Hippotraginae lineages. (B) Mutation sites in the 3D protein structures of three protein are indicated in green. The red chains represent the normal protein structure, and the blue chains represent those after parallel mutations. The locations where structural changes occurred before and after the mutation are indicated by arrows. (C) Expression level of liver in camel, cattle and human. t-test, * : $P \leq 0.05$; ** : $P \leq 0.01$; *** : $P \leq 0.001$.
