# Supplementary material for: INSIG1 parallel substitution drives lipid/sterol metabolic plasticity mediating desert adaptation in ungulates
Source: Commun Biol. 2026 Jan 12;9:245. doi: 10.1038/s42003-026-09523-z (PMC12905343; doi:10.1038/s42003-026-09523-z)
Supplement: Supplementary file 2 — Description of Additional Supplementary Files [file 42003_2026_9523_MOESM2_ESM.docx]

**Description of Additional Supplementary Files**File Name: Supplementary Data 1

Description: De novo genome assessment

File Name: Supplementary Data 2

Description: State of 18,430 ancestral placental mammal in Camelidae.

File Name: Supplementary Data 3

Description: Genome statistics for 22 ungulates used in this manuscript.

File Name: Supplementary Data 4

Description: The convergent positively selected genes (CPSGs) detected among Camelus and Hippotraginae lineages.

File Name: Supplementary Data 5

Description: The convergent GO terms of convergent positively selected genes between Camelus and Hippotraginae

File Name: Supplementary Data 6

Description: The convergent gene with parallel amino acid substitutions at conserved sites detected by PCOC, Conv_cal and CSUBST method with a series of standard in 25 pairwise comparison and 7 control groups

File Name: Supplementary Data 7

Description: The positive selected sites of convergent genes calculated by MEME

File Name: Supplementary Data 8

Description: The GO terms of convergent amino acid substitution genes among Camelus and Hippotraginae

File Name: Supplementary Data 9

Description: The GO terms of convergent amino acid substitution genes among control groups

File Name: Supplementary Data 10

Description: Untargeted metabolome data from camel serum

File Name: Supplementary Data 11

Description: Untargeted metabolome data from mouse serum

File Name: Supplementary Data 12

Description: Expression of INSIG1 and NPC1L1 in camels, cattle, and humans (FPKM)

File Name: Supplementary Data 13

Description: Co-Immunoprecipitation Antibody Information

File Name: Supplementary Data 14

Description: Relative ORO staining levels in the same groups showed increased neutral-lipid accumulation in cells expressing INSIG1(H150R) compared to wild-type INSIG1

File Name: Supplementary Data 15

Description: Relative DiI-LDL fluorescence levels were quantified in HepG2 cells transfected with empty vector (Con), INSIG1, or INSIG1(H150R)

File Name: Supplementary Data 16

Description: cholesterol and triglyceride concentrations in serum and liver of mutant and wild-type male mice after fasting for 14 hours

File Name: Supplementary Data 17

Description: The differentially expressed genes in liver and visceral fat of wildtype and male genome editing mice

File Name: Supplementary Data 18

Description: RT-qPCR quantitative results

File Name: Supplementary Data 19

Description: cholesterol and triglyceride concentrations in serum and liver of mutant and wild-type mice after fasting for 14 hours

File Name: Supplementary Data 20

Description: Untargeted metabolome data of liver from wildtype and genome editing mouse

File Name: Supplementary Data 21

Description: Bactrian transcriptome data for transcriptional annotation

File Name: Supplementary Data 22

Description: Relative mRNA expression of INSIG1

File Name: Supplementary Data 23

Description: The transcriptome sequencing data of the male edited mice

File Name: Supplementary Data 24

Description: Plasmid construction information
