# Supplementary material for: INSIG1 parallel substitution drives lipid/sterol metabolic plasticity mediating desert adaptation in ungulates
Source: Commun Biol. 2026 Jan 12;9:245. doi: 10.1038/s42003-026-09523-z (PMC12905343; doi:10.1038/s42003-026-09523-z)
Supplement: Supplementary file 4 — Reporting Summary [file 42003_2026_9523_MOESM4_ESM.pdf]

nature portfolio

Corresponding author(s): YuWang, Xia Bo,Chao Tong

Last updated by author(s): May 30, 2025

Reporting Summary

Nature Portfolio wishes to improve the reproducibility of the work that we publish. This form provides structure for consistency and transparency in reporting. For further information on Nature Portfolio policies, see our Editorial Policies and the Editorial Policy Checklist.

Statistics

For all statistical analyses, confirm that the following items are present in the figure legend, table legend, main text, or Methods section.

n/a	Confirmed	
☐	☒	The exact sample size (*n*) for each experimental group/condition, given as a discrete number and unit of measurement
☐	☒	A statement on whether measurements were taken from distinct samples or whether the same sample was measured repeatedly
☐	☒	The statistical test(s) used AND whether they are one- or two-sided<br>*Only common tests should be described solely by name; describe more complex techniques in the Methods section.*
☒	☐	A description of all covariates tested
☐	☒	A description of any assumptions or corrections, such as tests of normality and adjustment for multiple comparisons
☐	☒	A full description of the statistical parameters including central tendency (e.g. means) or other basic estimates (e.g. regression coefficient) AND variation (e.g. standard deviation) or associated estimates of uncertainty (e.g. confidence intervals)
☐	☒	For null hypothesis testing, the test statistic (e.g. *F*, *t*, *r*) with confidence intervals, effect sizes, degrees of freedom and *P* value noted<br>*Give P values as exact values whenever suitable.*
☒	☐	For Bayesian analysis, information on the choice of priors and Markov chain Monte Carlo settings
☒	☐	For hierarchical and complex designs, identification of the appropriate level for tests and full reporting of outcomes
☒	☐	Estimates of effect sizes (e.g. Cohen's *d*, Pearson's *r*), indicating how they were calculated

Our web collection on statistics for biologists contains articles on many of the points above.

Software and code

Policy information about availability of computer code

Data collection	Publicly available transcriptome data were downloaded, which comprising eight samples each of camels, cattle, and humans.<br>38 Bactrian transcriptome data for transcriptional annotation
Data analysis	BUSCO https://github.com/metashot/busco/blob/master/README.md<br>HiFiasm (Cheng and Concepcion et al., 2021) https://github.com/chhylp123/hifiasm/blob/master/README.md<br>Juicer (Durand and Shamim et al., 2016) https://github.com/aidenlab/juicer/blob/main/README.md<br>RepeatMasker(v4.1.1) (Tarailo-Graovac and Chen, 2009) https://github.com/Dfam-consortium/RepeatMasker/blob/master/README.md<br>TRF v4.09 (Benson, 1999) https://github.com/Benson-Genomics-Lab/TRF/blob/master/README.md<br>AUGUSTUS (Hoff and Stanke, 2013) https://github.com/Gaius-Augustus/Augustus/blob/master/README.md<br>Trinity (Haas and Papanicolaou et al., 2013) https://github.com/trinityrnaseq/trinityrnaseq/blob/master/README.md<br>EvidenceModeler (EVM) (Haas and Salzberg et al., 2008) https://github.com/EVidenceModeler/EVidenceModeler/blob/master/README.md<br>TOGA (Bogdan M. Kirilenko and Jebb et al., 2022) https://github.com/hillerlab/TOGA/blob/master/README.md<br>LAST (version last1205) (Kiełbasa and Wan et al., 2011) https://github.com/mcfrith/last-genome-alignments/blob/master/README.md<br>Multiz(Version 11.2) (Blanchette and Kent et al., 2004) https://github.com/multiz/multiz/blob/master/README.md<br>MACSE v2 (Ranwez and Douzery et al., 2018) https://github.com/ranwez/MACSE_V2_PIPELINES/blob/master/README.md<br>“Convergence at Conservative Sites” (CCS) (Xu, He, Guo, Zhang, Wyckoff, Greenberg, Wu and Shi, 2017) -<br>Gblocks (Talavera and Castresana, 2007) https://github.com/atmaivancevic/Gblocks<br>trimal (Capella-Gutiérrez and Silla-Martínez et al., 2009) https://github.com/inab/trimal<br>IQ-TREE (Nguyen and Schmidt et al., 2015) https://github.com/Cibiv/IQ-TREE

PAML (v4.9) (Yang, 2007) https://github.com/abacus-gene/paml
Hyphy (Kosakovsky Pond, Poon, Velazquez, Weaver, Hepler, Murrell, Shank, Magalis, Bouvier, Nekrutenko, Wisotsky, Spielman, Frost and Muse, 2020; Wertheim and Murrell et al., 2015) https://github.com/veg/hyphy
BUSTED-PH https://github.com/veg/hyphy-analyses/tree/master/BUSTED-PH https://github.com/veg/hyphy-analyses/tree/master/BUSTED-PH
KOBAS (Xie and Mao et al., 2011) https://github.com/xmao/kobas
Mixed Effects Model of Evolution (MEME) (Murrell, Wertheim, Moola, Weighill, Scheffler and Kosakovsky Pond, 2012) https://github.com/cinquin/MEME
"Convergence Event Counting and Probability Calculation" (Conv_cal) (Zou and Zhang, 2015) https://github.com/ztzou/conv_cal
CSUBST (Fukushima and Pollock, 2023) https://github.com/kfuku52/csubst
I-TASSR (Yang and Zhang, 2015; Zheng and Zhang et al., 2021; Zhou and Zheng et al., 2022) https://github.com/servehubco/I-TASSER
Alphafold2 (Jumper and Evans et al., 2021) https://github.com/lucidrains/alphafold2
ProtScale https://web.expasy.org/protscale/ https://gist.github.com/hryk/930826/b5b26bbfea74dc6d93a65c34ec21e684951b577f
FoldX (Schymkowitz and Borg et al., 2005) https://github.com/jbloomlab/pips-1.0/blob/master/src/foldx.py
MetaboAnalyst 5.0 (Pang and Chong et al., 2021) https://github.com/xia-lab/MetaboAnalystR
Trimmomatic (Bolger and Lohse et al., 2014) https://github.com/usadellab/Trimmomatic
HISAT2 (Kim and Paggi et al., 2019) https://github.com/DaehwanKimLab/hisat2
StringTie (Pertea and Pertea et al., 2015) https://github.com/gpertea/stringtie
DESeq2 (Love and Huber et al., 2014) https://github.com/thelovelab/DESeq2

For manuscripts utilizing custom algorithms or software that are central to the research but not yet described in published literature, software must be made available to editors and reviewers. We strongly encourage code deposition in a community repository (e.g. GitHub). See the Nature Portfolio guidelines for submitting code & software for further information.

Data

Policy information about availability of data

All manuscripts must include a data availability statement. This statement should provide the following information, where applicable:

- Accession codes, unique identifiers, or web links for publicly available datasets
- A description of any restrictions on data availability
- For clinical datasets or third party data, please ensure that the statement adheres to our policy

Genome sequencing was performed using CCS (Circular Consensus Sequencing Mode) on the Pacbio Sequel II platform. Finally, 37.59 Gb, 38.86 Gb, and 40.32 Gb of CCS reads in 3 cells were yielded, respectively. In addition, liver tissue collected from the same camel was used for Hi-C library construction, and 206.173 G raw data were obtained using the Illumina sequence.
We sequenced the transcriptome data from gene-edited and wild-type mice liver and visceral fat
The genome data generated in this study have been submitted to the NCBI BioProject database (https://www.ncbi.nlm.nih.gov/bioproject/) under accession number: BioProject ID: PRJNA1158569; BioSample ID: SAMN43543267.

Research involving human participants, their data, or biological material

Policy information about studies with human participants or human data. See also policy information about sex, gender (identity/presentation), and sexual orientation and race, ethnicity and racism.

Reporting on sex and gender	N/A
Reporting on race, ethnicity, or other socially relevant groupings	N/A
Population characteristics	N/A
Recruitment	N/A
Ethics oversight	N/A

Note that full information on the approval of the study protocol must also be provided in the manuscript.

Field-specific reporting

Please select the one below that is the best fit for your research. If you are not sure, read the appropriate sections before making your selection.

☒ Life sciences ☐ Behavioural & social sciences ☐ Ecological, evolutionary & environmental sciences

For a reference copy of the document with all sections, see nature.com/documents/nr-reporting-summary-flat.pdf

Life sciences study design

All studies must disclose on these points even when the disclosure is negative.

Sample size	500 ml blood from camel for hifi sequence

Sample size	liver tissue from camel for hic sequence<br>liver and fat transcriptome data from 3 gene-edited and 3 wild-type mice
Data exclusions	N/A
Replication	Liver and fat transcriptome data from 3 gene-edited and 3 wild-type mice
Randomization	N/A
Blinding	N/A

Reporting for specific materials, systems and methods

We require information from authors about some types of materials, experimental systems and methods used in many studies. Here, indicate whether each material, system or method listed is relevant to your study. If you are not sure if a list item applies to your research, read the appropriate section before selecting a response.

Materials & experimental systems

n/a	Involved in the study	
☒	☐	Antibodies
☐	☒	Eukaryotic cell lines
☒	☐	Palaeontology and archaeology
☐	☒	Animals and other organisms
☒	☐	Clinical data
☒	☐	Dual use research of concern
☒	☐	Plants

Methods

n/a	Involved in the study	
☒	☐	ChIP-seq
☒	☐	Flow cytometry
☒	☐	MRI-based neuroimaging

Eukaryotic cell lines

Policy information about cell lines and Sex and Gender in Research

Cell line source(s)	HepG2 cell was obtained from ATCC<br>Caco2 cell was gift from Beita Zhao (College of Food Science and Engineering, Northwest A&F University, Yangling, Shaanxi)
Authentication	N/A
Mycoplasma contamination	all cell lines tested negativate for mycoplasma
Commonly misidentified lines<br>(See ICLAC register)	N/A

Animals and other research organisms

Policy information about studies involving animals; ARRIVE guidelines recommended for reporting animal research, and Sex and Gender in Research

Laboratory animals	healthy female domestic Bactrian camel<br>C57BL/6J mice
Wild animals	N/A
Reporting on sex	N/A
Field-collected samples	N/A
Ethics oversight	Protocols used in the animal collection were approved by the guidelines of the<br>Northwest A&F University Animal Care Committee (Approval No. DK2022049).

Note that full information on the approval of the study protocol must also be provided in the manuscript.

Plants

Seed stocks	N/A
Novel plant genotypes	N/A
Authentication	N/A
